# Prototypical graph based deep label propagation with semantic augmentation for cross-subject and cross-session EEG emotion recognition

**DOI:** 10.3389/fnins.2026.1846592

**Published:** 2026-07-07

**Authors:** Yufang Dan, Li Zhu, Di Zhou

**Affiliations:** 1 School of Business Intelligence, Zhejiang Institute of Economics and Trade, Hangzhou, China; 2 Ningbo Key Laboratory of Aging Health Equipment and Service Technology, Ningbo, China; 3 Dazhou City Key Laboratory of Multidimensional Data Perception and Intelligent Information Processing, Sichuan University of Arts and Science, Dazhou, China; 4 Ningbo Key Laboratory of Intelligent Medical Chip and Equipment Technology, Ningbo, China; 5 School of Computer Science (School of Artificial Intelligence), Guangzhou Maritime University, Guangzhou, China; 6 Industrial Technological Institute of Intelligent Manufacturing, Sichuan University of Arts and Science, Sichuan China

**Keywords:** electroencephalography, emotion recognition, label propagation, prototypical learning, unsupervised domain adaptation

## Abstract

Electroencephalogram (EEG)-based emotion recognition faces significant generalization challenges in cross-subject and cross-session settings, primarily due to the inherent non-stationarity, individual differences, and semantic deficiency of EEG signals. To address these challenges and enhance the universality of affective brain-computer interface systems, this study proposes a novel unsupervised domain adaptation framework named Prototypical Graph-based Deep Label Propagation with Semantic Augmentation (PGDLP). PGDLP seamlessly integrates three core components—prototypical semantic augmentation, prototype-graph deep label propagation, and prototypical alignment—into an end-to-end optimized system. Specifically, class-wise multivariate normal distributions are constructed using source-domain feature statistics to augment target-domain features semantically, bridging the domain gap and mitigating semantic insufficiency. An adaptive similarity graph based on prototype-semantic distances is designed to optimize pseudo-label quality while reducing computational complexity. It is combined with linear projection and an exponential moving average (EMA) for dynamic refinement. Dual intra- and inter-domain alignment losses with an adaptive balance factor are introduced to enhance intra-class compactness and inter-domain transferability, thereby facilitating the learning of discriminative and domain-invariant features. Extensive experiments are conducted on three benchmark datasets (SEED, SEED-IV, DEAP) under four rigorous evaluation protocols (cross-subject cross-session, cross-subject single-session, within-subject cross-session, cross-database). Overall, PGDLP achieves superior recognition accuracy and generalization performance compared with most state-of-the-art methods across the majority of evaluation protocols. PGDLP also presents strong robustness against label noise, stable hyperparameter performance, and fast convergence. The results demonstrated that PGDLP outperforms state-of-the-art methods in recognition accuracy and generalization, with verified robustness to label noise, stable hyperparameters, and efficient convergence. This study provides a promising solution for unsupervised cross-domain EEG emotion recognition and offers valuable insights for domain adaptation research on other physiological signals.

## 1 Introduction

Electroencephalography (EEG) has become a cornerstone technology in affective computing, providing direct insights into the neural correlates of emotional states with unparalleled temporal resolution (Zhou et al., 2025; Si et al., 2023). Its applications span brain-computer interfaces (BCIs), mental health monitoring, and human-computer interaction, driving growing demand for robust EEG-based emotion recognition systems (Zhang et al., 2022b; Zheng and Lu, 2015). However, two intractable challenges hinder the practical deployment of such systems: the scarcity of labeled EEG data and the pervasive domain shift across subjects, sessions, or experimental settings (Ye et al., 2025; Zhang et al., 2022a). These challenges are exacerbated in unsupervised domain adaptation (UDA) scenarios, where target-domain labels are entirely unavailable, and only unlabeled target-domain data can be used (Li et al., 2022a, 2020).

Labeled EEG data acquisition is inherently resource-intensive, requiring expert annotation, strict experimental control, and prolonged data collection to align neural signals with subjective emotional states (Zhou et al., 2025; Zhong et al., 2020). Conversely, unlabeled EEG data is abundant yet underutilized by traditional supervised learning methods, which rely heavily on precise labels (Si et al., 2023; Berthelot et al., 2021). While semi-supervised learning (SSL) has made strides in integrating labeled and unlabeled data (Zhou et al., 2025; Dan et al., 2021), unsupervised domain adaptation imposes a more severe constraint by excluding target-domain labels entirely. Existing UDA methods for EEG emotion recognition, such as domain adversarial neural networks (DANN) (Ganin et al., 2016) and transfer component analysis (TCA) (Pan et al., 2011), focus primarily on aligning feature distributions between labeled source and unlabeled target domains but often fail to capture the intrinsic structural relationships of EEG data or fully exploit the semantic information latent in unlabeled samples (Berthelot et al., 2021; Peng et al., 2022).

Domain shift, caused by individual differences in brain anatomy, neural activity patterns, and EEG signal nonstationarity (Ye et al., 2025; Zheng and Lu, 2016), further degrades model generalization. Even state-of-the-art models trained on a source domain (e.g., a specific subject) often suffer significant performance degradation when applied to unseen target domains (e.g., new subjects) (He et al., 2022; Li et al., 2022). Traditional UDA methods address this by minimizing distribution discrepancies between source and target domains; however, they typically overlook two critical aspects: (1) the structural dependencies between EEG channels, which encode essential brain network information (Song et al., 2018; Jin et al., 2017), and (2) the semantic consistency of emotional representations across domains, which is crucial for maintaining discriminative power while aligning distributions (Zhou et al., 2024; Pinheiro, 2018). The above channel-oriented graph construction belongs to existing mainstream algorithms, while our proposed graph is built upon sample-prototype similarity rather than electrode spatial topology.

Recent advances in prototypical representation learning and graph neural networks (GNNs) have opened new avenues for addressing these limitations. Prototypical learning captures category-level semantic information, reducing reliance on individual labels and enhancing generalization (Zhou et al., 2024; Snell et al., 2017). Graph-based methods, on the other hand, model EEG channels as nodes in a graph, enabling effective modeling of spatial-temporal dependencies and structural features (Song et al., 2018; Jin et al., 2017). For example, EEGMatch (Zhou et al., 2025) integrated prototypical learning with multi-domain adaptation for semi-supervised cross-subject emotion recognition, while DS-AGC (Ye et al., 2025) proposed a dual-stream framework that combines graph contrastive learning and domain adaptation to capture both structural and non-structural features. PR-PL (Zhou et al., 2024) further demonstrated the efficacy of pairwise learning with prototypical representations in mitigating label dependence and individual differences. However, these methods either require labeled target data (in a semi-supervised setting) (Zhou et al., 2025; Ye et al., 2025) or lack effective mechanisms for semantic augmentation and label propagation in fully unsupervised settings (Zhou et al., 2024; Liu et al., 2022).

Another critical gap in existing UDA methods is the lack of semantic augmentation for unlabeled data. While data augmentation techniques such as EEG-Mixup (Zhou et al., 2025) and GAN-based synthesis (Zhang et al., 2022b) have improved model robustness in semi-supervised settings, they are rarely adapted to unsupervised domain adaptation, where semantic consistency across domains must be preserved without target labels. Additionally, label propagation methods, which infer pseudo-labels for unlabeled target data to guide adaptation (Peng et al., 2022; Chen et al., 2023), often suffer from noisy pseudo-labels in the absence of labeled target supervision, thereby limiting their effectiveness in UDA (Berthelot et al., 2021; Li et al., 2020).

To address these challenges, we propose a novel unsupervised domain adaptation framework, PGDLP (Prototypical Graph-based Deep Label Propagation with Semantic Augmentation), which integrates prototypical graph learning, deep label propagation, and semantic augmentation to achieve robust cross-domain EEG emotion recognition. The core contributions of this work are threefold:

(1) Propose a prototypical augmentation module: Construct a multivariate normal distribution from the class-wise feature means and intra-class covariance matrices of the source domain, and perform semantic augmentation on target domain features by sampling random directions per class. By dynamically adjusting the augmentation intensity coefficient (gradually increasing with training iterations), the impact of inaccurate prototype estimation in the early training stage is mitigated, effectively bridging the distribution gap between the source and target domains, addressing the semantic deficiency issue, and laying a high-quality feature foundation for subsequent label propagation and prototype alignment.
(2) Design a deep prototype-graph label propagation method: Construct an adaptive similarity graph based on prototype-semantic distances rather than traditional sample-to-sample distances, reducing computational complexity through matrix reconstruction; introduce a linear projection matrix to parameterize label prediction, mitigating overfitting to the similarity matrix; dynamically update prototypes and graph structures using an exponential moving average (EMA), iteratively optimizing pseudo-label quality, addressing noise accumulation in traditional label propagation, and improving the accuracy and stability of cross-domain label propagation.
(3) Extensive experiments on three benchmark EEG datasets (SEED, SEED-IV, and DEAP) under a cross-subject leave-one-subject-out protocol demonstrate that PGDLP outperforms state-of-the-art methods in handling distribution discrepancy and unreliable label propagation, achieving superior generalization with reduced computational costs.

The remainder of this study is organized as follows: Section 2 reviews related work. Section 3 details the proposed PGDLP framework. Section 4 presents experimental settings and results. Section 5 discusses performance and limitations. Section 6 concludes the study and outlines future directions.

## 2 Related work

### 2.1 EEG-based emotion recognition

Electroencephalography (EEG) has become a cornerstone of emotion recognition due to its ability to capture real-time neural activity with high temporal resolution, while avoiding the subjective bias associated with facial expressions or speech (Zhou et al., 2025; Si et al., 2023). Early studies focused on handcrafted features such as differential entropy (DE) (Duan et al., 2013) and brain network topology (Song et al., 2018), combined with traditional classifiers like support vector machines (SVM) (Suykens and Vandewalle, 1999) and random forests (RF) (Breiman, 2001), achieving promising results in subject-dependent scenarios (Zheng and Lu, 2015; Niu et al., 2023). With advances in deep learning, various neural network architectures have been proposed to model EEG's spatiotemporal characteristics. For example, dynamical graph convolutional neural networks (DGCNN) (Song et al., 2018) capture dynamic dependencies among EEG channels, while hierarchical spatiotemporal neural networks (Li et al., 2022a) integrate regional and global brain features to enhance discriminability. Recurrent neural networks (RNNs) and their variants (Zhang et al., 2019) have also been widely used to model temporal dynamics, demonstrating their effectiveness in capturing time-varying emotional responses (Zheng et al., 2018).

However, these methods often generalize poorly to cross-subject or cross-session tasks due to individual differences and the non-stationarity of the EEG signal (Rasoulzadeh et al., 2017; Zheng and Lu, 2016). To address this, recent works have explored multimodal fusion, (Liu et al., 2022), and channel attention mechanisms (Cui et al., 2020; Liu et al., 2020) to enhance feature robustness. For instance, regionally asymmetric convolutional neural networks (Cui et al., 2020) leverage hemispheric differences in emotional processing, while sparse autoencoders (Liu et al., 2020) extract compact discriminative features. Despite these advances, the reliance on sufficient labeled data and vulnerability to domain shift remain major barriers to practical deployment (Ye et al., 2025; Zhang et al., 2022a).

### 2.2 Unsupervised domain adaptation for EEG emotion recognition

Unsupervised domain adaptation (UDA) is a key approach to mitigating domain shift by aligning feature distributions between labeled source and unlabeled target domains (Pan et al., 2011; Ganin et al., 2016). Traditional UDA methods for EEG emotion recognition can be categorized into non-deep and deep learning-based approaches. Non-deep methods such as transfer component analysis (TCA) (Pan et al., 2011) and CORAL (Sun et al., 2016) aim to reduce distributional discrepancy through linear feature transformations, but they fail to model complex nonlinear dependencies in EEG signals (Jin et al., 2017). Kernel-based methods such as the geodesic flow kernel (GFK) (Gong et al., 2012) and subspace alignment (Fernando et al., 2013) improve nonlinear modeling but lack scalability to large-scale EEG datasets (Sun and Saenko, 2016).

Deep learning-based UDA methods have gained prominence due to their powerful feature learning capabilities. Domain adversarial neural networks (DANN) (Ganin et al., 2016) introduce adversarial training to learn domain-invariant features and have been extended to EEG tasks by multiple studies (Li et al., 2022, 2018). For example, bi-hemisphere DANN (Li et al., 2022) incorporates brain asymmetry to enhance adaptation, while deep adaptation networks (DAN) (Long et al., 2015a) use multiple kernel maximum mean discrepancy (MMD) for finer-grained distribution alignment. Generator-based methods (Huang et al., 2022; Li et al., 2022a) synthesize EEG samples to bridge domain gaps, preserving neurological information during adaptation. Graph-based UDA methods, such as domain adversarial graph attention models (DAGAM) (Xu et al., 2022) and regularized graph neural networks (RGNN) (Zhong et al., 2020), model EEG channels as graphs to capture structural dependencies, improving adaptation by leveraging spatial correlations (Song et al., 2018; Peng et al., 2022).

Nevertheless, existing UDA methods face critical limitations: (1) Most focus solely on aligning labeled source and target domains, ignoring unlabeled source data's potential to enhance feature generalization (Berthelot et al., 2021; Li et al., 2020); (2) They often overlook semantic consistency across domains, leading to inadequate feature discriminability after alignment (Zhou et al., 2024; Pinheiro, 2018); (3) Few methods integrate structural modeling of EEG channels with semantic augmentation, limiting their ability to capture both spatial dependencies and emotional semantics (Ye et al., 2025; Zhou et al., 2024).

#### 2.2.1 Prototypical representation and label propagation

Prototypical representation learning has emerged as an effective approach to reduce label dependence and enhance generalization by capturing category-level semantic information (Zhou et al., 2024; Snell et al., 2017). Prototypical networks (Snell et al., 2017) learn class centroids from labeled data, enabling few-shot learning and reducing sensitivity to individual differences. In EEG emotion recognition, PR-PL (Zhou et al., 2024) combines prototypical representation with pairwise learning to mitigate label noise and individual variability, achieving state-of-the-art performance on cross-subject tasks. EEGMatch (Zhou et al., 2025) further integrated prototypical learning with multi-domain adaptation and data augmentation, demonstrating the value of prototypical features in semi-supervised settings.

Label propagation (LP) is another key technique for UDA, inferring pseudo-labels for unlabeled target data to guide adaptation (Peng et al., 2022; Chen et al., 2023). Traditional LP methods use sample-based similarity graphs (Bao et al., 2018, 2022), which incur high computational costs and are vulnerable to noisy features (Berthelot et al., 2021). Graph-based LP methods (Peng et al., 2022; Xu et al., 2022) improve robustness by modeling channel dependencies, but they often suffer from static graph structures that fail to adapt to dynamic feature representations (Ye et al., 2025). Recent advances in LP, such as adaptive label propagation (Peng et al., 2022) and curriculum pseudo-labeling (Zhang et al., 2021), refine pseudo-labels using confidence thresholds. Yet, they lack effective semantic augmentation to ensure label consistency across domains (Chen et al., 2023).

#### 2.2.2 Semantic augmentation for EEG data

Data augmentation is crucial for addressing label scarcity and enhancing model robustness. Traditional methods such as Mixup (Zhang et al., 2018) and Gaussian noise injection (Zhang et al., 2022b) have been applied to EEG data, but they often ignore non-stationarity in EEG and structural characteristics (Zhou et al., 2025; Rasoulzadeh et al., 2017). Self-supervised augmentation frameworks such as GANSER (Zhang et al., 2022b) use generative adversarial networks (GANs) to synthesize high-quality EEG samples that preserve emotional semantics while enhancing diversity. EEG-Mixup (Zhou et al., 2025) improves upon standard Mixup by sampling valid IID-compliant EEG segments, ensuring augmented samples' validity.

However, semantic augmentation in UDA remains underexplored. Existing methods either focus on semi-supervised settings (Zhou et al., 2025; Zhang et al., 2022b) or lack explicit semantic alignment across domains (Zhou et al., 2024; Chen et al., 2021). Semantic augmentation for UDA requires preserving emotional semantics while aligning domain distributions, which is challenging without target labels (Berthelot et al., 2021; Pinheiro, 2018). Few frameworks integrate semantic augmentation with prototypical learning and label propagation, leaving a critical gap in leveraging the semantic potential of unlabeled data for cross-domain adaptation (Ye et al., 2025; Zhou et al., 2024).

To address these limitations, this study proposes a dynamic collaborative framework that unifies prototype-based semantic augmentation, deep prototype-graph label propagation, and prototype-guided domain registration, thereby mutually reinforcing these components to enhance cross-subject generalization.

## 3 Methodology

In this section, we formulate the *unsupervised domain adaptation* problem, discuss the classifier, the loss functions commonly used in prior work, and, finally, the transductive learning approach on which the PGDLP is based. In the experiments, we adopt a *convolutional neural network* (CNN)-based feature extractor to perform EEG emotion classification from preprocessed EEG feature matrices derived from multi-channel EEG signals, and we generalize the proposed formulation for physiological signal domain adaptation tasks.

### 3.1 Preliminary

#### 3.1.1 Problem formulation

We focus on the unsupervised domain adaptation (UDA) scenario, where we are given a labeled source domain $D_{s}$ denoted by $X_{s}={\left\{\left(x_{i}^{s},y_{i}^{s}\right)\right\}}_{i=1}^{n_{s}}\in {\mathbb{R}}^{d_{0}\times n_{s}}$ and an unlabeled target domain $D_{t}$ denoted by $X_{t}={\left\{\left(\left(x_{j}^{t},{ŷ}_{j}^{t}\right)\right)\right\}}_{j=1}^{n_{t}}\in {\mathbb{R}}^{d_{0}\times n_{t}}$, where $x_{i}^{s},x_{j}^{t}\in {\mathbb{R}}^{d_{0}}$ denote the *d*_0_-dimensional features of source and target samples, respectively, and *n*_*s*_ and *n*_*t*_ are the sizes of source and target datasets. Here, *X*_*s*_ are labeled according to $Y_{s}=\left(y_{1}^{s},\ldots ,y_{n_{s}}^{s}\right)$ with $y_{i}^{s}\in C$, where *C* = {1, …, *c*} is a discrete label set for *C* classes, and *X*_*t*_ are unlabeled with the corresponding pseudo-labels ${\hat{Y}}_{t}=({\hat{y}}_{1}^{t},\ldots ,{\hat{y}}_{n_{t}}^{t}$. We further let *X* = *X*_*s*_∪*X*_*t*_, *Y* = *Y*_*s*_∪*Y*_*t*_, and *n* = *n*_*s*_+*n*_*t*_.

The core challenge lies in domain shift–the marginal distributions of source and target domains are inconsistent, i.e., *P*_*s*_(*x*)≠*P*_*t*_(*x*), while the conditional distributions may also differ (*P*_*s*_(*y*|*x*)≠*P*_*t*_(*y*|*x*)), *x* ∈ *X*. The goal of UDA is to learn a classifier model that leverages labeled source data and unlabeled target data to generate reliable pseudo-labels for *X*_*t*_ and simultaneously learns domain-invariant discriminative features, thereby improving generalization performance in the target domain.

#### 3.1.2 Classifier

The network takes an input example from *X* and produces a vector of class confidence scores. We denote it by $f_{\theta }:X\to {\mathbb{R}}^{C}$, where θ are the network parameters. It is conceptually divided into two parts. The first is a feature-extraction network $\phi_{\theta }:X\to {\mathbb{R}}^{d}$ that maps the input to a feature vector or descriptor. We denote the descriptor of the *i*-th example *x*_*i*_ ∈ *X* by *v*_*i*_ = ϕ_θ_(*x*_*i*_), thus obtaining $V_{q}={\left\{v_{i}^{q}\right\}}_{i=1}^{n_{q}}=\phi_{\theta }\left(X_{q}\right)$, *q* ∈ {*s, t*}. We therefore let $V_{s}=\left(v_{1}^{s},\ldots ,v_{n_{s}}^{s}\right)$ be the descriptor set of *X*_*s*_ via deep network, where $v_{i}^{s}$ corresponds to $x_{i}^{s}$, and further let *V* = *V*_*s*_∪*V*_*t*_. The second typically consists of a *fully connected* (FC) layer applied on top of ϕ_θ_ and followed by a softmax layer, producing a vector of *confidence scores*. Function *f*_θ_ is the mapping from input space directly to confidence scores. The output of the network for the *i*-th example is *f*_θ_(*x*_*i*_) and the *prediction* is the one of maximum confidence score


$$\begin{array}{l} {ŷ}_{i}=\arg\underset{j}{\max}f_{\theta }{\left(x_{i}\right)}_{j}, \end{array}$$ (1)

where subscript *j* denotes the *j*-th dimension of the vector.

#### 3.1.3 Supervised loss

In supervised learning, the network is trained by minimizing a *supervised* loss term of the form


$$\begin{array}{l} L_{s}\left(X_{s},Y_{s};\theta \right)={\text{𝔼}}_{x_{i}\sim X_{s}}\left[\ell_{s}\left(f_{\theta }\left(x_{i}\right),y_{i}\right)\right], \end{array}$$ (2)

where *E*[.] is the *expectation* operator. Such a term is part of the total loss when training a network in a UDA setup (Shi et al., 2018; Tarvainen and Valpola, 2017). A standard choice for the loss function ℓ_*s*_ in classification is *cross-entropy*.

#### 3.1.4 Unsupervised loss

Pseudo-labeling is the process of assigning a pseudo-label ŷ_*i*_ to each example $x_{i}^{t}\in X_{t}$. With this strategy, the following additional *unsupervised* loss term applies


$$\begin{array}{l} L_{u}\left(X_{t},{Ŷ}_{t};\theta \right)={\text{𝔼}}_{x_{i}\sim X_{t}}\left[\ell_{u}\left(f_{\theta }\left(x_{i}\right),{ŷ}_{i}\right)\right], \end{array}$$ (3)

where again ℓ_*u*_ is any supervised loss function like cross-entropy. An example is the approach proposed by Lee (2013), who first trains a network *f*_θ_ with Equation 2 and then assigns pseudo-labels according to Equation 1 for *X*_*t*_.

Consistency loss is another common alternative in which the loss function can be applied to both labeled and unlabeled examples, encouraging consistency under different transformations of the data or the network. The so-called *consistency loss* (Tarvainen and Valpola, 2017; Shi et al., 2018) is defined as Equation 4:


$$\begin{array}{l} L_{con}\left(X;\theta \right)={\text{𝔼}}_{x_{i}\sim X_{t}}\left[\ell_{con}\left(f_{\theta }\left(x_{i}\right),f_{\tilde{\theta }}\left({\tilde{x}}_{i}\right)\right)\right] \end{array}$$ (4)

where ${\tilde{x}}_{i}$ refers to a different transformation of example *x*_*i*_ ∈ *X*_*t*_. ${\tilde{x}}_{i}$ remains input-space transformed sample. Note that according to the standard practice of data augmentation, every forward pass of *x*_*i*_ during training is performed under some random transformation. Parameter set $\tilde{\theta }$ is either equal to θ or any other transformation of it, such as a moving average over the sequence of network updates (Tarvainen and Valpola, 2017). A simple choice of ℓ_*con*_ is the squared Euclidean distance, i.e., $\ell_{con}\left(s,\tilde{s}\right)=||s-\tilde{s}{||}^{2}$ for $s,\tilde{s}\in {\mathbb{R}}^{C}$, forcing the two outputs to be as close as possible.

### 3.2 Proposed formulation

Below, we introduce prototypical networks (Snell et al., 2017), explain unsupervised prototype computation, prototype refinement, and inter-domain prototypical augmentation. Then, we present the Prototypical graph-based Label Propagation (PGDLP). Finally, we show how to optimize PGDLP by updating the prototypes, solving the partial assignment problem, and performing label propagation via linear projection. Moreover, we demonstrate how to obtain the final label prediction for the target set given learned prototypes.

**Figure 1:**
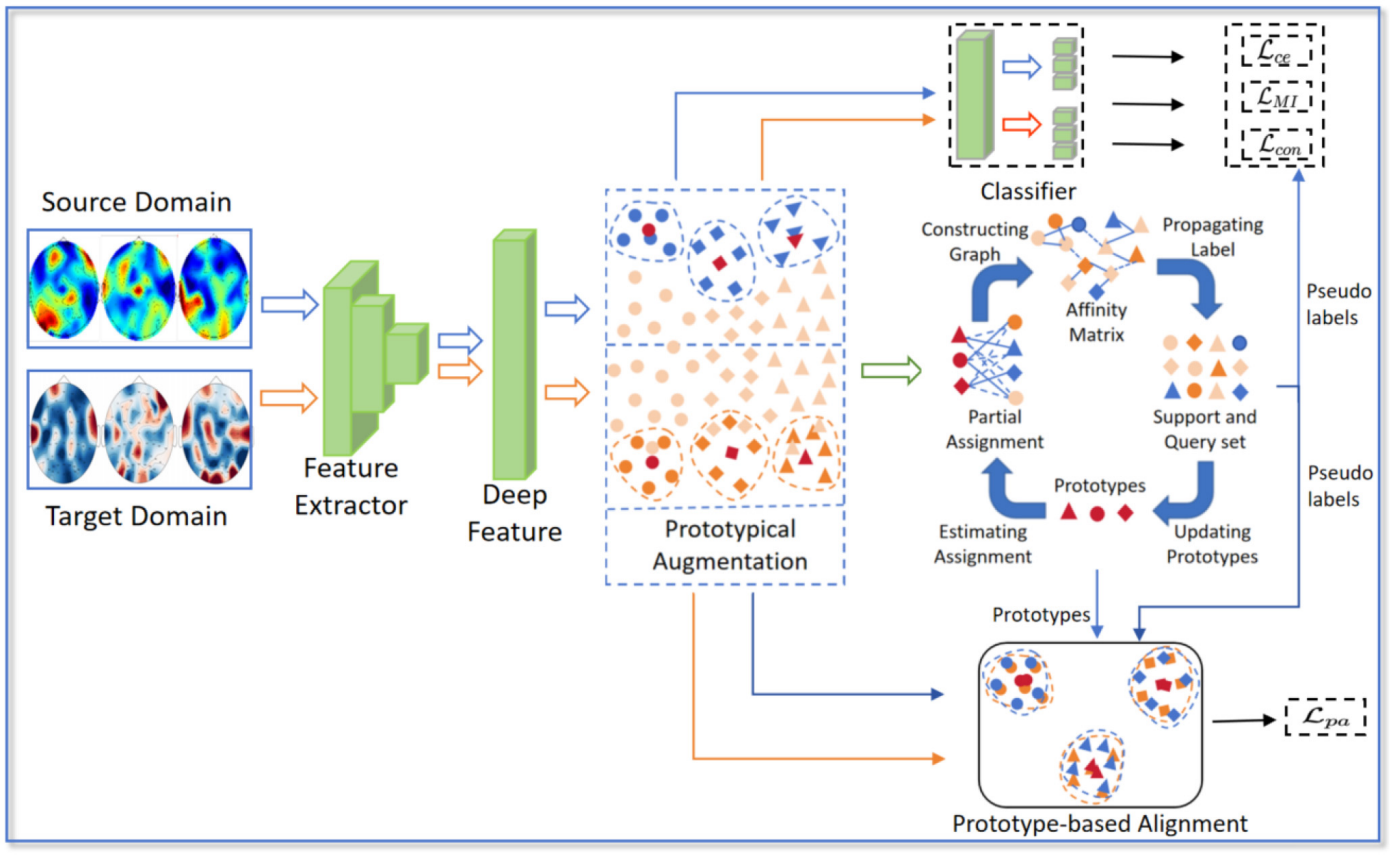
Overview of the proposed PGDLP framework. The framework integrates three tightly coupled core modules: prototypical semantic augmentation, prototype-graph deep label propagation, and adaptive prototype alignment. Following the alternating optimization rules in Algorithms 1, 2, the pipeline proceeds sequentially: shared feature extraction → target feature semantic augmentation → prototype graph construction and pseudo-label generation via label propagation → intra- and inter-domain prototype alignment for discriminative feature optimization, with mutual iterative feedback across all three modules throughout end-to-end training.

Figure 1 illustrates PGDLP.

#### 3.2.1 Prototypical augmentation

Previous work has demonstrated that translating features toward specific directions corresponds to meaningful semantic transformations when the features are mapped to the input space [9, 28, 10]. Specifically, there are many semantic directions in the deep feature space, such that translating a data sample in the feature space along one of these directions yields a feature representation corresponding to another sample with the same class prototype but different semantics.

This motivates us to augment the target domain by applying such semantic transformations on deep features. We hope that directions corresponding to meaningful transformations for each class are well represented by the principal components of the class's prototype. Based on this observation, we propose directly augmenting the target data in the feature space and integrating this procedure into the training of a deep model.

Specifically, to reduce intra- and inter-domain semantic deficiencies, we use Mixup (Zhang et al., 2018) to explore the structure between features and their corresponding class prototypes. The proposed prototype-oriented semantic data augmentation, referred to as prototypical augmentation (ProAug), has two important components: online estimation of class prototypes and class-conditional covariance matrices for the target, and augmentation via Mixup between class prototypes and features. The first component aims to find a distribution from which we can sample meaningful semantic transformation directions for data augmentation, while the second saves us from explicitly generating a large amount of extra training data. The model is then trained on these augmented target data to learn the optimal domain-invariant subspace for effective cross-domain label propagation.

Given that this semantic augmentation strategy relies heavily on the effective computation of class prototypes, the following section will first elaborate on the computation of domain-specific class prototypes, encompassing both prototype initialization and refinement.

#### 3.2.2 Computation of prototypes

Prototypical networks (Snell et al., 2017) compute a prototype of the *c*-th class as the mean vector of support samples belonging to this class:


$$\begin{array}{l} {\text{μ}}_{c}=\frac{1}{\left|X^{c}\right|}\underset{x_{i}:y\left(x_{i}\right)\in X^{c}}{\sum }v_{i}, \end{array}$$ (5)

where *v*_*i*_ = ϕ_θ_(*x*_*i*_), and *X*^*c*^⊂*X* is the set of examples labeled with class *c*.

Given a distance function Ω:ℝ^*d*^×ℝ^*d*^ → ℝ^+^, prototypical nets use Nearest Class Mean (NCM) to predict the label of sample *x* ∈ *X* by Equation 6:


$$\begin{array}{l} ŷ=\arg\underset{c}{\min}\text{Ω}\left(v_{i},{\text{μ}}_{c}\right). \end{array}$$ (6)

In the case of traditional DA, the prediction is performed independently on each episode. Thus, the mean vector depends only on the support set of *n*_*s*_ labeled examples, as shown in Equation 5, and is fixed for the given embedded features. However, in the case of the UDA, the prediction is performed inclusive of all target samples in *X*_*t*_.

#### 3.2.3 Refinement of prototypes

Transductive/semi-supervised Prototypical Network (Snell et al., 2017) treats prototypes μ_*c*_ in Equation 5 as clusters. The unlabeled samples $x_{i}^{t}$ are soft-assigned (Ding et al., 2018) to each cluster **μ**_*c*_, yielding *z*_*ic*_, whereas labeled samples use one-hot labels, i.e., $z_{ic}^{s}=1\text{for}c=y_{i}^{s}$ and $z_{ic}^{s}=0\text{for}c\neq y_{i}^{s}$. Specifically, refined prototypes are obtained as follows:


$$\begin{array}{l} \mu_{c}^{s}=\frac{\sum_{i=1}^{n_{s}}z_{ic}^{s}v_{i}^{s}}{\sum_{k=1}^{n_{s}}z_{kc}^{s}},\;\mu_{c}^{t}=\frac{\sum_{j=1}^{n_{t}}z_{jc}^{t}v_{j}^{t}}{\sum_{m=1}^{n_{t}}z_{mc}^{t}},\\ \mu_{c}=\frac{\sum_{i=1}^{n_{s}}z_{ic}^{s}v_{i}^{s}+\sum_{j=1}^{n_{t}}z_{jc}^{t}v_{j}^{t}}{\sum_{k=1}^{n_{s}}z_{kc}^{s}+\sum_{m=1}^{n_{t}}z_{mc}^{t}},\text{ where}\\ \;z_{ic}=\{\begin{array}{l} \frac{\exp\left(-\|v_{i}-\mu_{c}{\|}_{2}^{2}\right)}{\sum_{c^{\prime }}\exp\left(-\|v_{i}-\mu_{c^{\prime }}{\|}_{2}^{2}\right)}\text{ if }x_{i}\in X_{t}\\ \text{OneHot}(y_{i})\text{ if }x_{i}\in X_{s}. \end{array} \end{array}$$ (7)

where μ_*c*_ (*c* = 1, 2, ..., *C*) are the domain-agnostic prototypes, and *z*_*ic*_ is the (*i, c*)-th element of the matrix *Z*. The prediction of each query label follows Equation 6. Notice that although the prototype estimation leverages all data in the query set, the inference still depends only on prototypes and a single sample, rather than on prototypes and all samples.

We may use the gradient descent and the exponential moving average (EMA) to update ${\text{μ}}_{c}^{q}\;\left(q\in \left\{s,t\right\}\right)$ by Equation 8 to avoid instability that changing *Z* may pose:


$$\begin{array}{l} {\text{μ}}_{c}^{q}=\rho {\text{μ}}_{c}^{q\left(iter\right)}+\left(1-\rho \right){\text{μ}}_{c}^{q\left(iter-1\right)}, \end{array}$$ (8)

where the momentum term 0 ≤ ρ ≤ 1 controls the speed of adaptation of ${\text{μ}}_{c}^{q}$, and ${\text{μ}}_{c}^{q\left(iter\right)}$ and ${\text{μ}}_{c}^{q\left(iter-1\right)}$ are the prototypes of class *c* from the source(*s*)/target(*t*) domain at the current and previous iteration step *iter*, respectively.

** Remark 1**. To efficiently implement PGDLP in an end-to-end training manner, for each class *c*, we propose to estimate $\mu_{c}^{q}$ according to a memory module that stores all the latest features. To be concrete, we use a memory module to cache all features of two domains and update them in each batch. By doing so, we can discard those out-of-date features and replace them with the latest ones at negligible memory cost. Formally, in each iteration *iter*, we will update a batch of features and corresponding labels in the memory module *M* by Equation 9:


$$\begin{array}{l} v_{j}^{M}\leftarrow v_{j}^{\left(iter\right)},f_{j}^{M}\leftarrow f_{j}^{\left(iter\right)},j\in {\text{B}}^{\left(i\right)}, \end{array}$$ (9)

where *j* is the sample index within a batch **B**^(*i*)^ and $v_{j}^{M}/f_{j}^{M}$ is the feature/ (pseudo-)label stored in memory module *M*.

#### 3.2.4 Inter-domain prototypical augmentation

To tackle the inter-domain semantic deficiency, we need to perform meaningful, prototype-wise semantic augmentations across both domains.

To perform useful transformations, we enforce class-wise prototype alignment between target features and their corresponding class prototypes from the source domain, effectively bridging the overall semantic bias between the source and target domains. Specifically, for each class *c*, let $\mu_{c}^{s}$ and ${\text{Σ}}_{c}^{s}$ denote the estimated *c*-th class prototype vector and covariance matrix from the source domain, respectively. We exploit the intra-class covariance of source features to capture the source semantic variations. Specifically, we randomly sample semantic transformation directions from a multivariate distribution $N\left(\left(1-\text{λ}\right)\mu_{c}^{s},\;\epsilon {\text{Σ}}_{s}^{c}\right)$ for each class *c* from the target domain, where λ~Beta(α, α), for α ∈ (0, ∈ *fty*), and ϵ ∈ [0, 1] is a coefficient to control the strength of semantic data augmentation. Notably, $\left(1-\text{λ}\right)\mu_{c}^{s}$ attends to mitigating the overall semantic bias for class *c*, and ${\text{Σ}}_{c}^{s}$ focuses on providing abundant source intra-class semantic variation knowledge. By adding the sampled transformation vectors, the augmented inter-domain features will be close to source class prototypes and vary along source semantic variations.

During training, *C* class prototypes and covariance matrices are computed, one for each class. The augmented target feature ${\hat{v}}_{i}^{t\left(c\right)}$ for the *c*-th class is obtained by translating $v_{i}^{t\left(c\right)}=\phi_{\theta }\left(x_{i}^{t\left(c\right)}\right)\in \phi_{\theta }\left(X_{t}^{c}\right)$ along a random direction sampled from $N\left(\left(1-\text{λ}\right)\mu_{c}^{s},\;\epsilon {\text{Σ}}_{c}^{s}\right)$, where $X_{q}^{c}\left(q\in \left\{s,t\right\}\right)$ denotes the dataset belonging to the *c*-th class from the source (*q* = *s*) or target (*q* = *t*) domain. Equivalently, we have


$$\begin{array}{l} {\hat{v}}_{i}^{t\left(c\right)}\sim N\left(\text{λ}v_{i}^{t\left(c\right)}+\left(1-\text{λ}\right)\mu_{c}^{s},\epsilon {\text{Σ}}_{c}^{s}\right), \end{array}$$ (10)

As class prototypes and covariances are computed dynamically during training, the estimates in the first few epochs are not particularly informative when the network is not well trained. To address this issue, we let ϵ = (*iter*/*T*) × λ_0_, where *iter* and *T* are the current and maximum iterations respectively, and λ_0_ is a hyper-parameter. Consequently, as the training goes on, ϵ will gradually grow from 0 to λ0. And this sampling strategy will reduce the impact of less-accurate estimates of the mean and covariance at the start of training.

Considering a naive method, we can augment each $v_{i}^{t\left(c\right)}$ for *K* times via Equation 10 with its label preserved, which will result in an augmented feature set ${\hat{V}}_{t}^{c}$ = ${\left\{\left({\hat{v}}_{i1}^{t},{ỹ}_{i}^{t}\right),\left({\hat{v}}_{i2}^{t},{ỹ}_{i}^{t}\right),...,\left({\hat{v}}_{iK}^{t},{ỹ}_{i}^{t}\right)\right\}}_{i=1}^{n_{t}^{c}}$. The updating strategies of target feature set $\phi_{\theta }\left(X_{t}^{c}\right)$ is as follows:


$$\begin{array}{r} V_{t}^{c}=\phi_{\theta }\left(X_{t}^{c}\right)\leftarrow {\hat{V}}_{t}^{c}\cup \phi_{\theta }\left(X_{t}^{c}\right),\\ n_{t}^{c}\leftarrow \left(K+1\right)\times n_{t}^{c},\\ c=1,...,C. \end{array}$$ (11)

From Equation 11, we have the augmented target dataset $V_{t}={\left\{V_{t}^{c}\right\}}_{c=1}^{C}$, which can be exploited to train the UDA model.

Benefiting from the inherent statistical property of category prototypes, our proposed augmentation framework naturally mitigates adverse impacts from noisy early-stage pseudo-labels without additional confidence thresholding or entropy weighting operations. First, the category prototype is calculated via feature aggregation of intra-class samples, which is highly robust to a small number of outlier samples with incorrect pseudo-labels, as sporadic abnormal features barely shift the overall centroid of the prototype embedding. Second, the prototype aggregation mechanism treats all object categories equally when averaging features, free from distribution bias caused by imbalanced per-class sample counts arising from inaccurate pseudo-label allocation during early training. Therefore, our prototypical augmentation intrinsically constrains the propagation of pseudo-label noise throughout the whole training process and stabilizes the optimization of the UDA model.

#### 3.2.5 Label propagation on prototypical graph

Instead of training a generic classifier to classify new, unseen examples, the goal of transductive learning is to use *X* and *Y* to infer labels for examples in *X*_*t*_. In this study, we adopt the graph-based approach (Zhou et al., 2003) for transductive learning by label propagation (LP).

Naturally, a symmetric *adjacency matrix* *W* ∈ ℝ^*n*×*n*^ with zero diagonal can be constructed, whose elements *w*_*ij*_ are non-negative pairwise similarities between *v*_*i*_ ∈ *V* and *v*_*j*_ ∈ *V*. Its symmetrically normalized counterpart is given by *W* = *D*^−1/2^*WD*^−1/2^, where *D* = *diag*(*Wone*_*n*_) is the *degree matrix* and *one*_*n*_ is the all-ones *n*-vector, viz, its diagonal elements are given by $D_{ii}=\underset{j}{\sum }W_{ij}$. Naturally, we can construct a graph G=(V,E) where vertices *V* represent all labeled and unlabeled samples, and edges E are represented by the adjacency matrix *W*. We define a *n*×*C* *label matrix* Ŷ with elements by Equation 12:


$$\frac{1}{2}Tr({\hat{Y}}^{⊤}L\hat{Y})=\frac{1}{2}\sum_{i,j}W_{ij}{\left(\hat{y_{i}}-\hat{y_{j}}\right)}^{2}.$$ (12)

The graph Laplacian is then defined as *L* = *D*−*W*, which is used for smoothness-based regularization by taking into account the unlabeled data and then we have Equation 13:


$$\begin{array}{l} \frac{1}{2}Tr\left({Ŷ}^{⊤}LŶ\right)=\frac{1}{2}\underset{i,j}{\sum }W_{ij}{\left(\hat{y_{i}}-\hat{y_{j}}\right)}^{2}. \end{array}$$ (13)

For practical reasons, Zhou et al. (2003) are concerned not only with the smoothness but also with the impact of the supervised loss on the propagation. Thus, they minimize a combination of the smoothness and the squared error training loss:


$$\begin{array}{l} {Ŷ}^{*}=argmin_{Ŷ}\overset{n_{s}}{\underset{i=1}{\sum }}||{ŷ}_{i}-y_{i}^{s}{||}_{2}^{2}+\frac{{\text{λ}}_{M}}{2}Tr\left({Ŷ}^{⊤}LŶ\right), \end{array}$$ (14)

where λ_*M*_ is a regularization parameter.

Equation 14 relies on the quality of a fixed Laplacian matrix *L*, which largely determines the final performance of LP. Below, we introduce the Label Propagation on the prototypical graph (PGDLP) with an augmented target domain and source dataset. First, we parameterize the label propagation step and explain why. Then, we explain how to use prototypes to construct a graph, and we combine the above two components into PGDLP.

#### 3.2.6 Parameterized label prediction

Given the adjacency matrix *W*, we can solve label propagation by Equation 14. However, we pntroduce a linear projection *A* into the label propagation step to limit overfitting to the matrix *W*. Let:


$$\begin{array}{l} Ŷ=ZA, \end{array}$$ (15)

where $A={\left[a_{1},\cdots \;,a_{C}\right]}^{⊤}$ has *C* basis functions and *Z* comes from Equation 7 given a prototype set ${\left\{{\text{μ}}_{c}\right\}}_{c=1}^{C}$. Substitute Equation 15 into Equation 14, we obtain:


$$\begin{array}{l} A^{*}={\arg\min}_{A}||Z_{s}A-Y_{s}{||}_{F}^{2}+\frac{{\text{λ}}_{M}}{2}Tr\left(A^{⊤}Z^{⊤}LZA\right), \end{array}$$ (16)

where $Z_{s}={\left[z_{1}^{s},\ldots ,z_{n_{s}}^{s}\right]}^{⊤}\in {\mathbb{R}}^{n_{s}\times C}$, $Y_{s}={\left[y_{1}^{s},\ldots ,y_{n_{s}}^{s}\right]}^{⊤}\in {\mathbb{R}}^{n_{s}\times C}$ is the submatrix according to the assignment and label partition. Intuitively, we can regard *a*_*c*_ as a learnable label for the *c*-th prototype, which is non-sparse in contrast to a one-hot class vector. Based on the above model, One can estimate a soft score of the likely category of an inaccurate prototype.

#### 3.2.7 Prototype-based graph construction

There are two different graph definitions: (1) EEG channel graph: Nodes correspond to individual EEG electrodes, edge weights are determined by physical spatial connections of brain channels, which is widely adopted in conventional GNN-based EEG models; (2) Our proposed prototype graph: Nodes denote source/target deep feature samples plus category prototypes, edge affinity is calculated via sample-prototype semantic distance as formulated in Equations 17, 18, and no EEG electrode position information is incorporated during the adjacency matrix derivation. Prototype-based graphs are based on the idea that we can use a small number of prototypes to turn sample-to-sample affinity computations into much simpler sample-to-prototype affinity computations (Tian et al., 2023). Below, we explain how to construct a graph with prototypes. Given a prototype set ${\left\{{\text{μ}}_{c}\right\}}_{c=1}^{C}$, for each sample we obtain a partial assignment *z*_*j*_ ∈ *V* with soft assignment in Equation 7. We reconstruct the adjacency matrix *W* as:


$$\begin{array}{l} W=Z{\text{Λ}}^{-1}Z^{⊤}, \end{array}$$ (17)

where the diagonal matrix **Λ** ∈ ℝ^*C*×*C*^ is defined as $\Lambda_{cc}=\underset{i}{\sum }Z_{ic}$ (index *i* iterates over all samples). The corresponding Laplacian matrix is *L* = *I*−*Z***Λ**^−1^*Z*^⊤^. *W*_*ij*_ captures relation between the *i*-th and *j*-th samples by confounding variables **μ**_*c*_ according to the chain rule of Markov random walks:


$$\begin{array}{l} W_{ij}=p\left(v_{i}|v_{j}\right)=\overset{C}{\underset{c=1}{\sum }}p\left(v_{i}|{\text{μ}}_{c}\right)p\left({\text{μ}}_{c}|v_{j}\right)=p\left(v_{j}|v_{i}\right)\\ \;=\overset{C}{\underset{c=1}{\sum }}\frac{z_{jc}}{\underset{j^{\prime }}{\sum }z_{j^{\prime }k}}z_{i,k}=\overset{C}{\underset{c=1}{\sum }}\frac{z_{ic}z_{jc}}{{\text{Λ}}_{cc}}, \end{array}$$ (18)

where *p*(*v*_*i*_∣**μ**_*c*_) = *Z*_*ic*_ and *W*_*ij*_ = *W*_*ji*_. One may think of the above process as a 2-hop diffusion on a bipartite graph with samples *v*_*i*_ and prototypes **μ**_*c*_ located in two partitions of that graph. Notice the graph changes with prototypes.

#### 3.2.8 Prototype-based label propagation

Based on parameterized label prediction and prototype-based graph construction, we combine Equations 16, 17 into:


$$\begin{array}{l} \underset{A,Z,\;\mu }{\min}\frac{1}{2}||Z_{s}A-Y_{s}{||}_{F}^{2}+\frac{{\text{λ}}_{M}}{2}Tr\left(A^{⊤}Z^{⊤}\left(I-Z{\text{Λ}}^{-1}Z^{⊤}\right)ZA\right), \end{array}$$ (19)

where $\mu ={\left\{\mu_{c}\right\}}_{c=1}^{C}$ denotes the prototype set. As Equations 16, 17 are highly dependent on prototypes, instead of using the update of **μ**_*c*_ as in Equation 7, we use an alternative iterative step and use label prediction based on Equation 15 to update prototypes. First, we initialize each prototype as the mean vector of the support samples belonging to its class.

#### 3.2.9 Optimization

Below we explain how to optimize w.r.t. *Z, A* and μ by alternating. The order of optimization in each round is to minimize w.r.t. *Z*, then *A*, and finally μ.

Updating *Z*. First, given a prototype set $\mu ={\left\{\mu_{c}\right\}}_{c=1}^{C}$, we optimize the following equation w.r.t. *Z*:


$$\begin{array}{l} {\text{Z}}_{iter}=argmin_{Z}\underset{i,c}{\sum }z_{ic}||v_{i}-\mu_{c}{||}_{2}^{2},\text{s.t.}\underset{c}{\sum }z_{ic}=1. \end{array}$$ (20)

Equation 20 can be solved by Equation 7.

Updating *A*. Next, we solve Equation 19 w.r.t. *A* by globally-optimal closed-form formula:


$$\begin{array}{l} A_{iter}={\left(Z_{s}^{⊤}Z_{s}+{\text{λ}}_{M}Z_{iter}^{⊤}\left(I-Z_{iter}{\text{Λ}}^{-1}Z_{iter}^{⊤}\right)Z_{iter}\right)}^{-1}Z_{iter}^{⊤}Y. \end{array}$$ (21)

Subsequently, we can infer the label soft score by Equation 15, i.e., Ŷ_*iter*_ = *Z*_*iter*_*A*_*iter*_ is the output for updating prototypes in the next iteration. Substituting Equation 21 into Equation 15, we have:


$$\begin{array}{l} {Ỹ}_{iter}=Z_{iter}{\left(Z_{s}^{⊤}Z_{s}+{\text{λ}}_{M}Z_{iter}^{⊤}\left(I-Z_{iter}{\text{Λ}}^{-1}Z_{iter}^{⊤}\right)Z_{iter}\right)}^{-1}Z_{iter}^{⊤}Y. \end{array}$$ (22)

Using *A* is not mandatory, but this linear projection improves results by limiting overfitting during propagation.

Updating μ. We update μ by Equation 23:


$$\begin{array}{ll} \mu_{iter} & =argmin_{\mu }\underset{i,c}{\sum }{ŷ}_{ic}||v_{i}-\mu_{c}{||}_{2}^{2}, \end{array}$$ (23)

where one may set by Equation 24


$$\begin{array}{l} \mu_{c}=\frac{\sum_{i=1}^{n_{s}}v_{i}{ŷ}_{ic}+\sum_{j=1}^{n_{t}}v_{j}{ŷ}_{jc}}{\sum_{i^{\prime }=1}^{n_{s}}{ŷ}_{i^{\prime }c}+\sum_{j^{\prime }=1}^{n_{t}}{ŷ}_{j^{\prime }c}}. \end{array}$$ (24)

We use the gradient descent and the exponential running average to update μ by Equation 25 to avoid the instability that changing Ỹ may pose:


$$\begin{array}{l} \mu_{iter}=\left(1-\rho \right)\mu_{iter-1}+\rho {Ŷ}^{⊤}V, \end{array}$$ (25)

where ρ = 0.996 controls the speed of adaptation of μ_*iter*_.

Inference. For each query *v*_*i*_ ∈ *V*_*t*_, we predict its pseudo-label by $\underset{c\in \left\{1,\cdots \;,C\right\}}{\arg\max}{ŷ}_{jc}$ that corresponds to the maximum element of the *j*-th row of the resulting matrix Ŷ.

Algorithm 1 summarizes the standard LP on prototypical graph. Here, the so-called standard LP denotes the classic sample-wise label propagation algorithm originally proposed by Zhou et al. (2003), which constructs a similarity graph via pairwise sample distances and fixes the graph Laplacian throughout optimization (formulas (13)-(14)). Differently, the proposed ProtoLP is a modified variant built upon a prototype graph instead of raw sample affinity, hence it is not the conventional standard LP. Four steps indicated in italics are also indicated in Figure 1.

Algorithm 1 ProtoLP. 
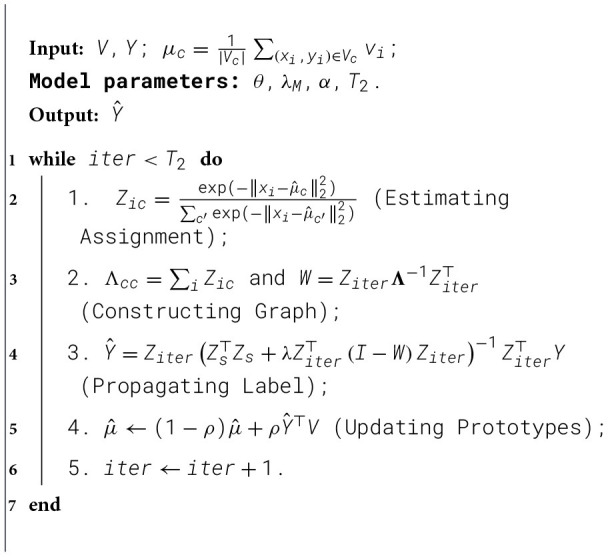

#### 3.2.10 Prototypical alignment network

For a single domain, ProtoNCE (Pinheiro, 2018) is proposed to learn the semantic structure of the data by iteratively clustering and learning representations, thereby driving features within the same cluster to become more aggregated and features in different clusters to become further apart. However, directly applying original ProtoNCE on raw EEG features may cause mis-clustering of EEG features with identical emotional labels but collected from distinct subjects/sessions, or clustering heterogeneous emotion EEG features from different domains into identical cluster centroids. To mitigate these problems, we propose to perform prototypical contrastive learning (Chen et al., 2021) separately in $D_{s}\cup D_{t}$. Specifically, once prototypes are obtained, we construct prototype-based distribution alignment, including intra-domain and Inter-domain alignment, to enhance intra-class compactness and inter-class separation in the source and target domains, respectively, thereby leading to discriminative, domain-invariant feature learning. We explain only on the source domain for succinct notation; all operations are performed on the target similarly.

#### 3.2.11 Intra-domain alignment

We perform intra-domain alignment for the target domain. Specifically, for a given feature, we construct a positive pair consisting of the feature and its corresponding class prototype, and a negative pair with a prototype from a different class. We use the standard ProNCE (Pinheiro, 2018) loss for prototypical contrastive learning. For the target domain, this loss for a given feature $\phi \left(x_{i}\right)\in \phi \left(X_{t}^{c}\right)$ with prototype $\mu_{c}^{t}$ is defined as by Equation 26:


$$\begin{array}{l} L_{intra}^{t}=\underset{x_{i}\in X_{t}^{c}}{\sum }-\log\frac{\exp\left(\phi \left(x_{i}\right)\cdot \mu_{c}^{t}/\tau_{c}^{t}\right)}{\exp\left(\phi \left(x_{i}\right)\cdot \mu_{c}^{t}/\tau_{c}^{t}\right)+\underset{k\neq c}{\sum }\exp\left(\phi \left(x_{i}\right)\cdot \mu_{k}^{t}/\tau_{c}^{t}\right)}, \end{array}$$

$$\begin{array}{l} c=1,...,C \end{array}$$ (26)

where $\tau_{c}^{t}$ is the clustering concentration factor for the *c*th class from target domain.

Similarly for source domain, we define $L_{intra}^{s}$. We sum these losses are to get the intra-domain alignment loss as by Equation 27:


$$\begin{array}{l} L_{intra}=L_{intra}^{t}+L_{intra}^{s} \end{array}$$ (27)

#### 3.2.12 Inter-domain alignment

We also align features across source and target domains. Specifically, we align features from the source domain with prototypes from the target domain in an asymmetric manner. Given a feature $\phi \left(x_{i}^{s}\right)$ from the source domain, we construct a positive pair with the prototype from the same class from the target domain and a negative pair with the prototype from a different class in the target domain. The inter-domain alignment loss for source feature $\phi \left(x_{i}\right)\in \phi \left(X_{s}^{c}\right)$ with target prototype $\mu_{c}^{t}$ is defined as, by Equation 28


$$\begin{array}{l} L_{inst}=\underset{x_{i}\in X_{s}^{c}}{\sum }\\ -\log\frac{\exp\left(\phi \left(x_{i}\right)\cdot \mu_{c}^{t}/\tau_{c}^{t}\right)}{\exp\left(\phi \left(x_{i}\right)\cdot \mu_{c}^{t}/\tau_{c}^{t}\right)+\underset{k\neq c}{\sum }\exp\left(\phi \left(x_{i}\right)\cdot \mu_{k}^{t}/\tau_{c}^{t}\right)},\\ c=1,...,C \end{array}$$ (28)

Moreover, the model aims to maximize the similarity between the prototypes in the source and target domains. In the prototypical contrastive learning, $\mu_{c}^{s}$ and $\mu_{c}^{t}$ form the positive pair while the remaining prototypes from both domains form the negative pairs. The remaining prototypes from both domains are pushed apart in the latent space. This is achieved through employing a modified NT-Xent (normalized temperature-scaled cross-entropy) contrastive loss (Pan et al., 2019) for domain adaptation, given by Equation 29:


$$\begin{array}{l} L_{proto}\left(\mu_{c}^{t},\mu_{c}^{s}\right)=-\log\frac{h\left(\mu_{c}^{t},\mu_{c}^{s}\right)}{h\left(\mu_{c}^{t},\mu_{c}^{s}\right)+\overset{C}{\underset{\begin{array}{c} r=1\\ q\in \left\{s,t\right\} \end{array}}{\sum }}1_{\left\{r\neq c\right\}}h\left(\mu_{c}^{t},\mu_{r}^{q}\right)},\\ c=1,...,C \end{array}$$ (29)

where $h\left(\text{u},\text{v}\right)=\exp\left(\frac{{\text{u}}^{⊤}\text{v}}{||\text{u}{||}_{2}||\text{v}{||}_{\text{2}}}/\tau_{c}\right)$ measures the exponential of cosine similarity, *1* is an indicator function and τ_*c*_ is the temperature hyperparameter.

To sum up these two terms, we have the following inter-domain alignment loss Equation 30:


$$\begin{array}{l} L_{inter}=L_{inst}+L_{proto} \end{array}$$ (30)

Note that $L_{Intra}$ aims to enforce the discriminative clustering of intra-class features from the source and target domains, and $L_{Inter}$ aligns the class prototypes belonging to the same class across domains. Thus, via simultaneously minimizing $L_{Intra}$ and $L_{Inter}$, the PGDLP addresses both the objectives of discriminativity and transferability (Chen et al., 2019). Intuitively, domain features are more likely to be assigned to prototypes that correspond to dominant classes or are closer to them (or both).

In the nutshell, by introducing a balancing parameter α and combining $L_{intra}$ and $L_{inter}$, we forms the following prototype alignment problem by Equation 31:


$$\begin{array}{l} L_{pa}=\alpha L_{Intra}+\left(1-\alpha \right)L_{Inter}, \end{array}$$ (31)

where α ∈ [0, 1] is a balance factor between intra-domain discriminativity and inter-domain transferability. Intrinsically, as α approaches 0, it signifies a significant disparity in the distribution between the two domains, thus underscoring the paramount importance of inter-domain prototype alignment. Conversely, when α tends toward 1, it implies a relatively major divergence in intra-domain cluster structures, thus leading to the so-called “class collision” phenomenon. In practical scenarios, intra-domain discriminativity and inter-domain adaptivity typically contribute differently to domain adaptation performance. By determining the optimal balance factor α, PGDLP can be tailored to address a diverse array of domain adaptation challenges, as discussed in the Discussion.

### 3.3 Alternating optimization algorithm

#### 3.3.1 Supervised cross-entropy loss

Inferring pseudo-labels from matrix *Z* by hard assignment has two undesired effects: first, we define pseudo-labels on all unlabeled examples, while we do not have the same certainty for each example. Second, pseudo-labels may not be balanced over classes, which will impede learning.

To address the former issue, we associate each pseudo-label with a weight that reflects the certainty of the prediction. We use *entropy*, as a measure of uncertainty, to assign weight ${\hat{\sigma }}_{i}$ to example *x*_*i*_ ∈ *X*, defined by Equation 32:


$$\begin{array}{l} {\hat{\sigma }}_{i}=1-\frac{H\left({ẑ}_{i}\right)}{\log\left(C\right)}, \end{array}$$ (32)

where Ẑ is the row-wise normalized counterpart of *Z*, i.e., ${ẑ}_{ij}=z_{ij}/\underset{c}{\sum }z_{ic}$, and function *H*:ℝ^*C*^ → ℝ is the entropy function. Weight ${\hat{\sigma }}_{i}$ is normalized in [0, 1] because log(*C*) is the maximum possible entropy in ℝ^*C*^.

To deal with the latter issue of class imbalance, we assign a weight ζ_*c*_ to class *c* that is inversely proportional to class population, defined as $\zeta_{c}={\left(\left|n_{s}^{c}|+|n_{t}^{c}\right|\right)}^{-1}$, where $n_{s}^{c}$ (resp. $n_{t}^{c}$) are the examples labeled (resp. pseudo-labeled) as class *c*.

Given the above definitions of per-example and per-class weights, we associate the following *weighted loss* to the labeled and pseudo-labeled examples


$$\begin{array}{l} L_{ce}\left(X,Y_{s},{Ŷ}_{t};\theta \right)=\frac{1}{n_{s}}\overset{n_{s}}{\underset{i=1}{\sum }}\zeta_{y_{i}^{s}}\ell_{s}\left(f_{\theta }\left(x_{i}^{s}\right),y_{i}^{s}\right)\\ \;+\frac{1}{n_{t}}\overset{n_{t}}{\underset{i=1}{\sum }}{\hat{\sigma }}_{i}\zeta_{{\hat{y^{t}}}_{i}}\ell_{u}\left(f_{\theta }\left(x_{i}^{t}\right),{ŷ}_{i}^{t}\right), \end{array}$$ (33)

which is the sum of weighted versions of Equations 2, 3, where ℓ_*s*_ (ℓ_*u*_) is the standard *cross-entropy* loss function. In contrast to Equation 3, pseudo-labels originate in LP rather than network predictions.

#### 3.3.2 Mutual information maximization

In information theory, mutual information *I*(*X*; *Y*) measures how related two random variables *X* and *Y* are. Actually, strong correlations between target features and predictions will benefit the semantic augmentations, because the extracted features will be more informative and contain more important semantics for predictions, ignoring trivial semantics. Thus, we employ mutual information maximization on the target data, i.e., minimizing the loss in Equation 34.


$$\begin{array}{l} L_{MI}=\overset{C}{\underset{c=1}{\sum }}{\hat{P}}^{c}\log{\hat{P}}^{c}-\frac{1}{n_{t}}\overset{n_{t}}{\underset{j=1}{\sum }}\overset{C}{\underset{c=1}{\sum }}{\text{p}}_{jc}^{t}\log{\text{p}}_{jc}^{t}, \end{array}$$ (34)

where ${\text{p}}_{j}^{t}=f_{\theta }\left(x_{j}^{t}\right)$, ${\hat{P}}^{c}=\frac{1}{n_{t}}\overset{n_{t}}{\underset{j=1}{\sum }}P_{jc}^{t}$. Since the target domain is unlabeled, we use the average of target predictions to approximate the ground-truth distribution on the target domain.

#### 3.3.3 Iterative training

As the PGDLP can introduce (possibly) better pseudo labels of target instances, we train the deep network ψ for discriminative feature representations with the pseudo-labeled target instances and source labeled ones.


$$\begin{array}{l} L=L_{ce}+{\text{λ}}_{A}L_{pa}+\gamma L_{con}+\beta L_{MI},\\ L_{con}=\underset{x_{i}^{t}\sim X_{t}}{\sum }\left[1\left(\max\left({ŷ}_{i}^{t}\right)\geq \delta \right)\ell_{con}\left(f_{\theta }\left(x_{i}^{t}\right),f_{\theta }\left(v_{i}^{t}\right)\right)\right], \end{array}$$ (35)

where ℓ_*con*_ is the squared Euclidean distance, the target domain is supervised with predicted results from the proposed label propagation approach, and $\max\left(\overset{t}{\underset{i}{ŷ}}\right)$ returns the highest possibility score with a predefined threshold value δ. $v_{i}^{t}$ remains input-space transformed sample.

Since the discriminative feature leaning of deep network would in turn benefit the construction of affinity matrix of the prototypical graph, we therefore alternate in obtaining (possibly) better pseudo labels of target instances via PGDLP, and using the obtained pseudo-labeled target instances (together with the labeled source ones) to train the DA network with loss in Equation 35.

Specifically, given the augmented target features and the source dataset, we construct a prototypical graph, perform label propagation, and compute the network training losses; we then integrate these components into an iterative learning process.

We begin by randomly initializing the network parameters θ, and we train the network for *T*_1_ epochs in a fully supervised manner on the *n* labeled/pseudo-labeled examples *X* using the supervised loss term (Equation 33).

The trained network then provides the starting point for the following iterative process. First, we extract descriptors *V* from the entire training set *X* and compute class prototypes from both domains for the augmented target features. Second, we compute an adjacency matrix *W* to construct a prototypical graph based on source and augmented target domains. Third, we perform label propagation on the prototypical graph by solving the linear system (Equation 19) and assign pseudo-labels to unlabeled examples *X*_*t*_ by Equation 22. We repeat this iterative process for *T*_2_ steps. Finally, we train the network for one epoch on the entire training set *X* using Equation 35. We repeat this iterative process for *M* epochs. The above is summarized in Algorithm 2.

Algorithm 2 Alternating algorithm of PGDLP. 
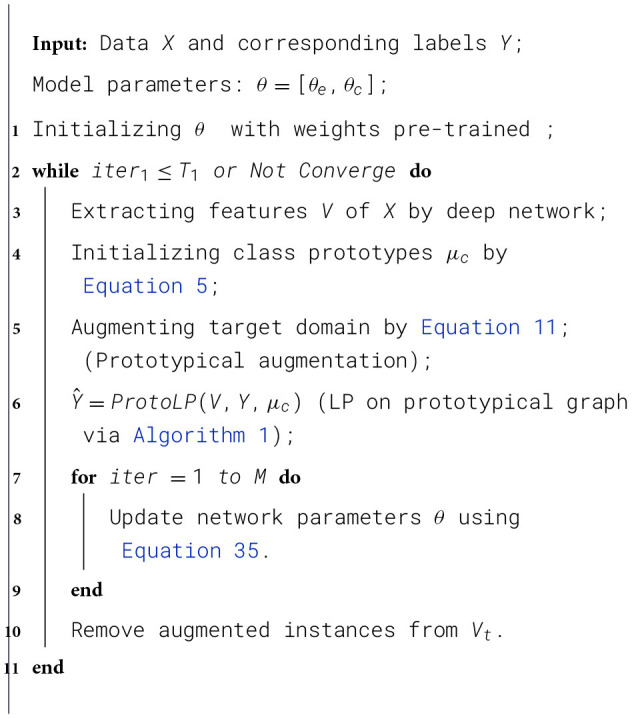

#### 3.3.4 Target inference

For each sample $x_{j}^{t}\in X_{t}$, we predict its pseudo-label by $\underset{c\in \left\{1,\cdots \;,C\right\}}{\arg\max}{\text{f}}_{jc}^{t}$ that corresponds to the maximum element of the *j*-th row of the resulting matrix *F*_*t*_.

Intuitively, we can regard *a*_*c*_ as a learnable label for the *c*-th prototype, which is non-sparse in contrast to a one-hot class vector. Additionally, for the *i*th instance $x_{i}^{t}\in X_{t}$, we also can regard ***Z***_*ic*_ as its pseudo-label with respect to the *c*-th category, Notice that although the prototypes estimation leverages all data in the same cluster, the inference of ***Z***_*ic*_ still only depends on prototypes and a single sample rather than prototypes and all samples.

## 4 Discussion

### 4.1 Balance factor for prototype alignment

To theoretically understand the idea, we introduce the domain adaptation theory proposed by Shai et al. (2007), which reveals the ingredients of target generalization error ϵ_*t*_. Formally, let H denote the hypothesis space and h∈H denote the classifier, we can formulate the upper bound of ϵ_*t*_ as:


$$\begin{array}{l} \epsilon_{t}\left(h\right)\leq \epsilon_{s}\left(h\right)+\frac{1}{2}d_{H\Delta H}\left(D_{s},D_{t}\right)+{\text{λ}}^{*},\forall h\in H \end{array}$$ (36)

where ϵ_***s***_(*h*) is the source generalization error of *h*, $d_{H\Delta H}\left(S,T\right)$ is the HΔH -distance between source and target domains, and ${\text{λ}}^{*}=\epsilon_{s}\left(h^{*}\right)+\epsilon_{t}\left(h^{*}\right)$ denotes the error of an ideal joint hypothesis h^*^ on source and target domains. With the supervision of labeled source data, ϵ_*s*_(*h*) is well bounded. Besides, $L_{inter}$ can fill the domain gap, the proposed method further bounds the $d_{H\Delta H}\left(D_{s},D_{t}\right)$. Moreover, theoretically, the PGDLP can generate infinite augmented target features that are close to source prototypes class-wisely, enabling the source classifier to jointly minimize $\epsilon_{s}\left(h^{*}\right)$ and $\epsilon_{t}\left(h^{*}\right)$ of the shared error λ^*^ on the augmented training set. To sum up, the proposed method aligns well with the theory, thereby further enhancing transferability and Discriminability.

Notably, PGDLP theoretically requires minimizing all three components in Equation 36 to constrain the upper bound of the expected error in the target domain. For example, although the inter-domain prototypical alignment $L_{Inter}$ may decrease $d_{H\Delta H}\left(D_{s},D_{t}\right)$, it can inadvertently compromise discriminability and render the data indistinguishable, ultimately causing λ^*^ to increase. In the subsequent subsection, we adopt a adaptive weighting strategy for domain alignment and class discrimination learning, guaranteeing the concurrent reduction of both $d_{H\Delta H}\left(D_{s},D_{t}\right)$ and λ^*^. To accomplish this, we introduce a novel concept involving the adaptive interplay between HΔH divergence and the combination error λ^*^, contrasting with the isolated training methodologies employed in prior models. This innovative technique successfully diminishes the upper bound of ϵ_*t*_(*h*) in this study.

Specifically, we explore computing the adaptive alignment factor (α) to facilitate an easy, dynamic, and quantitative evaluation of the relative importance of the $L_{Intra}$ and $L_{Inter}$. This factor can be adaptively updated within the network itself. First, we use deep representations to initialize α, thereby enhancing the robustness and accuracy of PGDLP. Second, PGDLP automatically fine-tunes the adaptive factor (α) by directly using the prototypical alignment losses, making the process simpler and more efficient. More specifically, we make the first attempt toward calculating α (i.e., $\hat{\alpha }$) by exploiting the intra-domain and inter-domain semantic structures. We adopt the A-distance (Si et al., 2023) as the basic measurement. Formally, the intra-domain A-distance and the inter-domain A-distance can be respectively defined as


$$\begin{array}{l} d_{A}^{Intra}\left(D_{s},D_{t}\right)=2\left(1-2L_{clu}^{Intra}\right),\;d_{A}^{Inter}\left(D_{s},D_{t}\right)=2\left(1-2L_{clu}^{Inter}\right). \end{array}$$ (37)

Therefore, the overall domain discrepancy can be covered from Equation 37. We then estimate the adaptive prototypical alignment factor $\hat{\alpha }$ using the following formulation Equation 38:


$$\begin{array}{l} \hat{\alpha }=\frac{d_{A}^{Intra}\left(D_{s},D_{t}\right)}{d_{A}^{Intra}\left(D_{s},D_{t}\right)+d_{A}^{Inter}\left(D_{s},D_{t}\right)}. \end{array}$$ (38)

To ensure robustness, this estimate must be computed at each iteration of the dynamic distribution adaptation process executed by Algorithm 2, since the intra-domain feature distribution may change after each evaluation of the inter-domain prototypical distribution. In practical scenarios, it is feasible to initialize α as 0 in the initial epoch. Subsequently, pseudo-labels for the target domain can be obtained after each epoch. The adaptive prototypical alignment factor α can then be calculated at the end of each epoch. Ultimately, as the training process converges, PGDLP will acquire a highly robust adaptive prototypical alignment factor $\hat{\alpha }$.

### 4.2 Clustering concentration estimation

Intuitively, the embedded features surrounding each prototype may demonstrate varying levels of concentration. We employ $\tau_{c}^{s/t}$ (*c* = 1, 2, ..., *K*) as an estimator for the concentration level of samples within the *c*-th class. A smaller value of $\tau_{c}^{s/t}$ indicates a lower dispersion of samples around the prototype, or conversely, a higher degree of concentration. In this context, features ${\left\{v_{i}^{s/t\left(c\right)}\right\}}_{i=1}^{n_{c}^{s/t}}$, which belong to the same cluster as the prototype $\mu_{c}^{s/t}$, are utilized to calculate $\tau_{c}^{s/t}$. When the average distance between $v_{i}^{s/t}$ and $\mu_{c}^{s/t}$ is minimal, and the cluster comprises a significant number of feature points (indicating that $n_{c}^{s/t}$ is large), it follows that $\tau_{c}^{s/t}$ ought to be small, signifying high concentration. Therefore, $\tau_{c}^{s/t}$ can be defined as by Equation 39:


$$\tau_{c}^{s/t}=\frac{\sum_{j=1}^{n_{c}^{s/t}}{\left\|v_{j}^{s/t}-\mu_{c}^{s/t}\right\|}_{2}^{2}}{n_{c}^{t}\log(n_{c}^{s/t}+\delta_{c})},c=1,2,...,C,$$ (39)

where the smoothing parameter $\delta_{c}=\frac{1}{\sqrt{2*\pi }}\exp^{-|n_{c}^{s/t}|}$ serves to guarantee that smaller clusters do not yield excessively high values for $\tau_{c}^{s/t}$.

In the loss $L_{intra}$, $\tau_{c}^{s/t}$ serves as a scaling factor, adjusting the similarity measure between the embedded feature $v_{i}^{s/t}$ and its corresponding prototype $\mu_{c}^{s/t}$. In clusters with larger $\tau_{c}^{s/t}$ values, indicating looser structures, the similarity between the feature and the prototype is comparatively low. Through appropriate regulation of $\tau_{c}^{s/t}$, minimizing the loss $L_{Intra}$ facilitates the alignment of embedded features closer to their prototypes. Conversely, in compact clusters (with smaller $\tau_{c}^{s/t}$), the embedded features and the prototype are more similar, so it is unnecessary to move the features closer to the prototype. Hence, the joint minimization of $L_{Intra}$ and $L_{Inter}$ leads to a more balanced clustering structure. This effectively mitigates the risk of converging on a trivial solution in which a majority of embedded features are assigned to a single cluster.

## 5 Experimental analysis

Three widely recognized benchmark datasets—SEED (Zheng and Lu, 2015; Duan et al., 2013), SEED-IV (Zheng et al., 2018; Zheng and Lu, 2016; Zhou et al., 2025), and DEAP (Sander et al., 2012)—are utilized in this section to comprehensively evaluate the model's effectiveness in EEG-based emotion recognition.

### 5.1 Datasets

#### 5.1.1 Description

The SEED dataset contains EEG-based emotional data collected from 15 subjects who watched 15 movie clips tailored to induce three distinct emotional states: positive, negative, and neutral. In contrast, the SEED-IV dataset also recruits 15 participants and employs 24 movie segments to evoke four emotions: happiness, sadness, neutrality, and fear. Both datasets involve three experimental sessions conducted on separate days, with a 1-week interval between consecutive sessions, and EEG signals were acquired using a 62-channel ESI Neuroscan system.

Additionally, we aim to investigate the model's performance in cross-dataset scenarios, particularly exploring whether acceptable recognition accuracy can be maintained when training and testing data come from different subjects, are recorded with distinct EEG devices, and involve emotional states triggered by diverse stimuli. Further, we seek to assess whether multi-source domain adaptation methods can enhance performance under such conditions. To this end, we leverage the publicly accessible DEAP dataset (Sander et al., 2012) for emotional state analysis. This dataset includes data from 32 subjects who watched 40 1-min music videos designed to elicit emotional responses, with their physiological signals recorded simultaneously. After viewing each video, participants rated their emotional experiences across five dimensions: valence (pleasantness), arousal (excitement level), dominance (degree of control), liking (personal preference), and familiarity (stimulus recognition). Ratings range from 1 (lowest) to 9 (highest) across all dimensions except familiarity, which is scored from 1 to 5.

For a more comprehensive understanding of these three benchmark datasets, refer to Lan et al. (2019). As noted in Zhong et al. (2020) and Lan et al. (2019), significant differences exist among these datasets, which may arise from multiple factors such as variations in experimental sessions, participant demographics, experimental protocols, EEG recording equipment, and types of emotional stimuli.

#### 5.1.2 Feature extraction

Preprocessing of EEG data from the SEED dataset adhered to established protocols outlined in Luo et al. (2024). Initially, EEG signals were downsampled to 200 Hz, followed by artifact removal, including Electrooculogram (EOG) and Electromyography (EMG) interference. A 0.3–50 Hz bandpass filter was then applied to enhance signal integrity, and each trial was segmented into 1–s intervals. Given that SEED trial durations ranged from 185 to 265 s (dependent on the emotional stimulus duration), all trials were truncated to a uniform 185 s to ensure inter-class data consistency.

For the DEAP dataset, EEG signals were recorded using Biosemi ActiveTwo devices at an initial sampling rate of 512 Hz, which was later downsampled to 128 Hz. Emotional ratings in DEAP are originally on a five-point scale; to align with the SEED dataset's emotion categorization, we discretized the valence dimension into positive (valence > 7), neutral (3 ≤ valence ≤ 7), and negative (valence < 3). Trials where most participants confirmed successful emotion induction were selected: Trial 18 (positive, 27 participants), Trial 16 (neutral, 28 participants), and Trial 38 (negative, 19 participants). Only data from 14 participants (Subjects 2, 5, 10, 11, 12, 13, 14, 15, 19, 22, 24, 26, 28, 31) who consistently reported valid emotion elicitation in these trials were retained. Each DEAP trial lasts 63 s, with the first 3 s designated as baseline (no emotion induction); thus, the valid segment starts at the 4th second, resulting in a 60-s effective trial length. This trial and subject filtering strategy strictly follows the dataset preprocessing rules from Lan et al. (2019); Zhong et al. (2020).

To capture emotion-relevant information, differential entropy (DE) features (Zhong et al., 2020; Lan et al., 2019) were computed across five frequency bands: Delta (1–3 Hz), Theta (4–7 Hz), Alpha (8–13 Hz), Beta (14–30 Hz), and Gamma (31–50 Hz). This process generated 310-dimensional features (62 channels × 5 frequency bands) per 1-s segment for the SEED dataset, with each trial contributing 185 such samples. For the DEAP dataset, the feature vector was 160-dimensional (32 channels × 5 frequency bands), and each valid trial contained 60 samples, which were used as inputs to the model.

### 5.2 Experimental protocols

In line with the methodology described in Donahue et al. (2014), the proposed PGDLP method can be efficiently trained using deep features derived from pre-established models. Specifically, we fine-tune pre-trained deep architectures such as ResNet-101—where EEG differential entropy features (62 channels × 5 bands) are reshaped into 62 × 5 matrices to mimic image-like inputs for ResNet-101—on the source domain before extracting deep representations from EEG signals (He et al., 2016). The Bottleneck layer outputs of ResNet-101 are leveraged to balance semantic and spatial information for effective cross-domain alignment. Pre-experimental results confirmed that ResNet-101's deep residual structure outperforms ResNet-50 and DenseNet in cross-domain scenarios (Donahue et al., 2014; Yan et al., 2025), with well-developed open-source implementations supporting reproducibility. ResNet-101 is uniformly adopted as the backbone feature extractor across all experiments. These extracted deep features are subsequently utilized to train the emotion recognition model. Experiments ran on four NVIDIA A100 GPUs using PyTorch.

Given that hyperparameter tuning remains a persistent challenge in machine learning, this study empirically explores the parameter space to identify optimal configurations. The PGDLP framework involves five core hyperparameters, initialized as λ_*M*_ = 0.1, λ_*A*_ = 0.6, γ = 1, and β = 1. We select hyper-parameter λ_0_ from 0.1, 0.25, 0.5, 0.75, 1.0 and found λ_0_ = 0.25 works well on all datasets. To ensure fair comparison, all baseline methods and PGDLP use the same backbone, optimizer, learning rate schedule, batch size, and training epochs following the standard UDA protocol (Ganin et al., 2016). The learning rate is set as $\eta_{\text{iter}}=\eta_{0}{\left(1+\beta_{0}\cdot \text{iter}\right)}^{-\delta }$, where η_0_ = 0.01, β_0_ = 0.0002, δ = 0.75. Mini-batch stochastic gradient descent (SGD) is employed for optimization, with the momentum coefficient set to 0.9. The batch size is configured to 32 for the source domain and 64 for the target domain. The total number of training epochs is fixed at 500. A fixed random seed is set and kept unchanged throughout the experimental procedure. Each experimental setup is repeatedly run 10 times to calculate the mean accuracy and standard deviation.

To comprehensively evaluate the robustness and consistency of the proposed PGDLP method and enable fair comparison with existing works, four distinct validation protocols incorporating diverse evaluation strategies are adopted, as referenced in (Zhou et al. 2024); (Tao et al. 2024):

1) Cross-sUbject Cross-sEssion (CUCE) with Leave-One-Subject-Out (LOSO) cross-validation: This rigorous protocol aims to assess the model's ability to generalize to unseen subjects and sessions. In each iteration, the session data of one subject is designated as the target domain, while session data from all other subjects serve as the source domain. The process is repeated until every subject's session data has been used as the target once. Owing to inherent individual differences and session-related environmental variations, this protocol poses a stringent test for EEG-based emotion recognition models.
2) Cross-sUbject Single-sEssion (CUSE) with LOSO cross-validation: As a widely adopted validation technique in EEG emotion recognition (Li et al., 2019a; Luo et al., 2018; Li et al., 2019b; Zhou et al., 2024; Luo et al., 2024), this method assigns single-session data from one subject as the target, with data from all other subjects used as the source. The iterative training and validation process continues until each subject has been designated as the target once, and only the first session is considered, consistent with standard research practices.
3) Within-sUbject Cross-sEssion (WUCE) with LOSO cross-validation: Aligned with prevalent time-series cross-validation approaches, this protocol utilizes historical data to predict current or future data. For each subject, the first two sessions are assigned to the source domain, and the subsequent session acts as the target domain. Final results are reported as the mean accuracy and standard deviation across all subjects.
4) Cross Database Cross-Validation (CDCV): Following the configurations outlined in (Tao and Dan 2021); (Tao et al. 2023), 32 common channels from the SEED and DEAP datasets are used to construct a unified 160-dimensional feature space, enhancing cross-dataset generalization. Six cross-dataset adaptation scenarios are formulated: DEAP → SI, DEAP → SII, DEAP → SIII, SI → DEAP, SII → DEAP, and SIII → DEAP (where SI, SII, SIII denote SEED's Session I, II, III, respectively). When DEAP serves as the source, 2,520 data points are selected; 2,775 data points are chosen from each SEED session as the target. Conversely, when each SEED session is the source, 41,625 resampled data points are used for training, with 180 samples from DEAP as the target.

### 5.3 Experimental results

The experimental results are presented as the mean performance across all subjects. For other deep benchmarks, we directly use their publicly available source code to fine-tune the pre-trained models used in their respective works.

#### 5.3.1 CUCE with LOSO cross-validation

To assess the effectiveness and consistency of the PGDLP across cross-subject and cross-session contexts, we employ LOSO cross-validation to evaluate the proposed PGDLP approach within the CUCE framework, using both the SEED and SEED-IV datasets. CUCE imposes a rigorous test of the model's generalization ability, requiring it to overcome the dual challenges of individual differences among subjects and environmental variation across experimental sessions. The results are presented in Tables 1, 2.

**Table 1:** The mean accuracies (%) and standard deviations (%) of CUCE with LOSO cross-validation on the SEED database.

Methods	Pacc	Methods	Pacc
Traditional machine learning methods			
RF (Breiman, 2001)	69.60 ± 7.64*	KNN (Duda et al., 2000)	60.66 ± 7.93*
SVM (Vapnik, 1995)	62.24 ± 5.48*	Adaboost (Zhu et al., 2006)	71.87 ± 5.70*
TCA (Pan et al., 2011)	65.31 ± 6.04*	CORAL (Sun et al., 2016)	69.22 ± 4.11*
SA (Fernando et al., 2013)	61.41 ± 9.75*	GFK (Gong et al., 2012)	67.36 ± 6.52*
DICE (Liang et al., 2019)	73.56 ± 4.23*	GAKT (Ding et al., 2018)	74.82 ± 7.14*
MDDD (Luo et al., 2024)	76.60 ± 6.79*	EDPC (Dan et al., 2024)	76.82 ± 6.14*
JAGP (Peng et al., 2022)	75.64 ± 3.14*	JTSR (Peng et al., 2023)	76.30 ± 5.12*
Deep learning methods			
PARSE (Zhang et al., 2022a)	82.44 ± 5.00*	DAN (Long et al., 2019)	82.51 ± 3.71*
DDC (Tzeng et al., 2014)	82.17 ± 4.96*	DANN (Ganin et al., 2016)	84.79 ± 6.44*
PR-PL (Zhou et al., 2024)	85.56 ± 4.78*	DADPc (Dan et al., 2025)	87.83 ± 4.21 -
DLP-NNM (Wang et al., 2025)	88.23 ± 7.13 -	RGNN (Zhong et al., 2020)	84.56 ± 6.20*
DAPLP (Zhong et al., 2025)	89.48 ± 6.44 -	UDDA (Li et al., 2022b)	86.12 ± 7.36 -
PGCN (Jin et al., 2024)	83.10 ± 4.02*	DS-AGC (Ye et al., 2025)	84.94 ± 6.71*
GCPL (Li et al., 2024)	82.88 ± 6.70*	EEGMatch (Zhou et al., 2025)	86.30 ± 5.04*
PGDLP(Ours)	**91.65** **±5.16**		

Best in bold.^*^The performance of PGDLP is statistically significant compared with other methods at *p*-value ≤ 0.05.

**Table 2:** The mean accuracies (%) and standard deviations (%) of CUCE with LOSO cross-validation on the SEED-IV database.

Methods	Pacc	Methods	Pacc
Traditional machine learning methods			
KNN (Duda et al., 2000)	40.83 ± 7.28*	SVM (Vapnik, 1995)	51.78 ± 12.85*
Adaboost (Zhu et al., 2006)	53.44 ± 9.12*	TCA (Pan et al., 2011)	56.56 ± 13.77*
CORAL (Sun et al., 2016)	49.44 ± 9.09*	SA (Fernando et al., 2013)	64.44 ± 9.46*
GFK (Gong et al., 2012)	45.89 ± 8.27*	KPCA (Schölkopf et al., 1998)	51.76 ± 12.89*
DICE (Liang et al., 2019)	66.75 ± 7.25*	GAKT (Ding et al., 2018)	64.48 ± 5.52*
MDDD (Luo et al., 2024)	64.90 ± 10.25*	EDPC (Dan et al., 2024)	67.88 ± 5.21*
JAGP (Peng et al., 2022)	66.47 ± 7.53*	JTSR (Peng et al., 2023)	67.83 ± 8.06*
Deep learning methods			
DLP-NNM (Wang et al., 2025)	76.78 ± 6.08 -	DAN (Long et al., 2019)	58.87 ± 8.13*
RGNN (Zhong et al., 2020)	73.84 ± 8.02*	BiHDM (Li et al., 2019c)	69.03 ± 8.66*
BiDANN (Li et al., 2022)	65.59 ± 10.39*	DANN (Ganin et al., 2016)	54.63 ± 8.03*
PR-PL (Zhou et al., 2024)	74.92 ± 7.92 -	PARSE (Zhang et al., 2022a)	69.78 ± 8.22*
DDC (Tzeng et al., 2014)	72.17 ± 5.86*	DADPc (Dan et al., 2025)	75.31 ± 6.22 -
DAPLP (Zhong et al., 2025)	78.17 ± 5.04 -	UDDA (Li et al., 2022b)	77.62 ± 6.30 -
PGCN (Jin et al., 2024)	76.41 ± 7.46 -	DS-AGC (Ye et al., 2025)	77.81 ± 7.26 -
GCPL (Li et al., 2024)	72.51 ± 8.35*	EEGMatch (Zhou et al., 2025)	73.60 ± 7.53*
PGDLP(Ours)	**79.58** **±6.27**		

Best in bold. ^*^The performance of PGDLP is statistically significant compared with other methods at *p*-value ≤ 0.05.

Traditional machine learning methods exhibit generally low overall performance. Compared to the highest accuracy of 76.82%±6.14% in the three-class SEED task, the highest accuracy in SEED-IV (a four-class task) is 67.88%±5.21%. The increase in the number of classes elevates the difficulty of feature discrimination, resulting in an overall accuracy lower than that of the SEED dataset. Deep learning methods significantly outperform traditional ones, but their performance on SEED-IV is generally lower than on SEED, indicating limitations in the reliability of pseudo-labels and the effectiveness of feature alignment in multi-class scenarios.

Algorithm 2 achieves optimal performance on the SEED dataset with an accuracy of 91.65%±5.16%(Table 1), which is 2.17% higher than the best baseline and 14.83% higher than the highest value of traditional methods. This demonstrates that PGDLP's integrated framework of prototype-based semantic augmentation, prototype-graph label propagation, and prototype alignment can effectively address the non-stationarity and semantic deficiency of EEG signals in cross-subject and cross-session scenarios, while maintaining high recognition accuracy and generalization ability under complex individual and environmental variations. PGDLP still ranks first on the SEED-IV dataset with an accuracy of 79.58% ± 6.27%(Table 2), which is 1.41% higher than the best baseline and 11.7% higher than the highest value of traditional methods. This indicates that PGDLP's prototype-based design can effectively enhance inter-class discriminability in multi-class scenarios. Even as the number of classes increases and feature overlap rises, cross-domain label propagation can still improve accuracy through dynamic prototype optimization and semantic augmentation.

#### 5.3.2 CUSE with LOSO cross-validation

Tables 3, 4 summarize the experimental outcomes from the LOSO recognition task conducted on the SEED and SEED-IV datasets within the CUSE framework, along with a benchmarking against previous studies.

**Table 3:** The mean accuracies (%) and standard deviations (%) of CUSE with LOSO cross-validation on the SEED database.

Methods	Pacc	Methods	Pacc
Traditional machine learning methods			
TKL (Long et al., 2015b)	63.54 ± 15.47*	T-SVM (Ronan et al., 2006)	68.57 ± 9.54*
TCA (Pan et al., 2011)	63.64 ± 14.88*	TPT (Zheng and Lu, 2016)	73.86 ± 11.05*
KPCA (Schölkopf et al., 1998)	61.28 ± 14.62*	GFK (Gong et al., 2012)	71.31 ± 14.09*
SA (Fernando et al., 2013)	66.00 ± 10.89*	DICA (Ma et al., 2019)	69.40 ± 7.80*
DBN (Zheng and Lu, 2015)	61.01 ± 12.38*	SVM (Vapnik, 1995)	58.18 ± 13.85*
DICE (Liang et al., 2019)	74.22 ± 7.33*	GAKT (Ding et al., 2018)	72.29 ± 4.66*
MDDD (Luo et al., 2024)	84.57 ± 9.49*	EDPC (Dan et al., 2024)	82.34 ± 4.52*
JAGP (Peng et al., 2022)	77.07 ± 7.10*	JTSR (Peng et al., 2023)	80.89 ± 7.66*
Deep learning methods			
PARSE (Zhang et al., 2022a)	82.11 ± 5.83*	DAN (Long et al., 2019)	83.81 ± 8.56*
RGNN (Zhong et al., 2020)	85.30 ± 6.72*	BiHDM (Li et al., 2019c)	85.40 ± 7.53*
WGAN-GP (Luo et al., 2018)	87.10 ± 7.10*	DLP-NNM (Wang et al., 2025)	89.71 ± 10.39*
ATDD-DANN (Du et al., 2022)	90.92 ± 1.05*	JDA-Net (Li et al., 2019b)	88.28 ± 11.44 *
R2G-STNN (Li et al., 2022a)	84.16 ± 7.63*	SimNet (Pinheiro, 2018)	81.58 ± 5.11*
BiDANN (Li et al., 2022)	83.28 ± 9.60*	DResNet (Ma et al., 2019)	85.30 ± 8.00*
ADA (Philip et al., 2017)	84.47 ± 10.65*	DANN (Ganin et al., 2016)	81.65 ± 9.92*
PR-PL (Zhou et al., 2024)	93.06 ± 5.12*	DADPc (Dan et al., 2025)	93.58 ± 6.35 -
DAPLP (Zhong et al., 2025)	89.44 ± 4.89*	UDDA (Li et al., 2022b)	88.10 ± 6.54*
PGCN (Jin et al., 2024)	84.59 ± 8.68*	DS-AGC (Ye et al., 2025)	86.38 ± 7.25*
GCPL (Li et al., 2024)	80.74 ± 6.05	EEGMatch (Zhou et al., 2025)	92.45 ± 6.85*
PGDLP(Ours)	**96.67** **±2.25**		

Best in bold. ^*^The performance of PGDLP is statistically significant compared with other methods at *p*-value ≤ 0.05.

**Table 4:** The mean accuracies (%) and standard deviations (%) of CUSE with LOSO cross-validation on the SEED-IV database.

Methods	Pacc	Methods	Pacc
Traditional machine learning methods			
TKL (Long et al., 2015b)	63.54 ± 15.47*	T-SVM (Ronan et al., 2006)	68.57 ± 9.54*
TCA (Pan et al., 2011)	63.64 ± 14.88*	TPT (Zheng and Lu, 2016)	73.86 ± 11.05*
KPCA (Schölkopf et al., 1998)	61.28 ± 14.62*	GFK (Gong et al., 2012)	71.31 ± 14.09*
SA (Fernando et al., 2013)	66.00 ± 10.89*	DICA (Ma et al., 2019)	69.40 ± 7.80*
DBN (Zheng and Lu, 2015)	61.01 ± 12.38*	SVM (Vapnik, 1995)	58.18 ± 13.85*
DICE (Liang et al., 2019)	74.22 ± 7.33*	GAKT (Ding et al., 2018)	72.29 ± 4.66*
MDDD (Luo et al., 2024)	76.60 ± 6.79*	EDPC (Dan et al., 2024)	76.82 ± 7.14*
JAGP (Peng et al., 2022)	76.54 ± 9.50*	JTSR (Peng et al., 2023)	77.48 ± 7.01*
Deep learning methods			
PARSE (Zhang et al., 2022a)	82.11 ± 5.83*	DAN (Long et al., 2019)	83.81 ± 8.56*
RGNN (Zhong et al., 2020)	85.30 ± 6.72*	BiHDM (Li et al., 2019c)	85.40 ± 7.53*
WGAN-GP (Luo et al., 2018)	87.10 ± 7.10*	DLP-NNM (Wang et al., 2025)	94.15 ± 9.33 -
ATDD-DANN (Du et al., 2022)	90.92 ± 1.05*	JDA-Net (Li et al., 2019b)	88.28 ± 11.44*
R2G-STNN (Li et al., 2022a)	84.16 ± 7.63*	SimNet (Pinheiro, 2018)	81.58 ± 5.11*
BiDANN (Li et al., 2022)	83.28 ± 9.60*	DResNet (Ma et al., 2019)	85.30 ± 8.00*
ADA (Philip et al., 2017)	84.47 ± 10.65*	DANN (Ganin et al., 2016)	81.65 ± 9.92*
PR-PL (Zhou et al., 2024)	93.06 ± 5.12 -	DADPc (Dan et al., 2025)	94.62 ± 4.37 -
DAPLP (Zhong et al., 2025)	94.68 ± 6.18 -	UDDA (Li et al., 2022b)	93.14 ± 9.50 -
PGCN (Jin et al., 2024)	90.07 ± 7.16*	DS-AGC (Ye et al., 2025)	86.46 ± 10.53*
GCPL (Li et al., 2024)	84.72 ± 8.18*	EEGMatch (Zhou et al., 2025)	92.45 ± 06.85 -
PGDLP(Ours)	**95.78** **±3.41**		

Best in bold. ^*^The performance of PGDLP is statistically significant compared with other methods at *p*-value ≤ 0.05.

CUSE considers only data from a single session, eliminating environmental variation between sessions and requiring only the resolution of individual differences among subjects. Consequently, the overall accuracy is significantly higher than that in the CUCE scenario. As observed in Table 3, deep learning methods exhibit excellent performance, with mainstream algorithms such as PR-PL (93.06%±5.12%), DADPc (93.58% ± 6.35%), and EEGMatch (92.45% ± 6.85%) all achieving an accuracy of over 90%. The proposed PGDLP algorithm achieves a substantially higher accuracy of 96.67% ± 11.25%, which is 3.09% higher than the best baseline and 14.33% higher than the highest value among traditional methods. This result verifies that in scenarios where only individual differences among subjects exist, PGDLP's prototype-graph label propagation can significantly reduce computational complexity while improving the reliability of pseudo-labels. It effectively mines cross-subject shared semantic information among EEG features, achieving higher recognition accuracy.

As shown in Table 4, Algorithm 2 achieves optimal performance with an accuracy of 95.78%±9.41%, which is 1.1% higher than the best baseline. Notably, PGDLP's performance improvement is more stable in the complex four-class scenario than in the three-class scenario, demonstrating that its prototype alignment and semantic augmentation mechanisms are more adaptable at optimizing the discriminability of multi-class features and are suitable for more refined emotion category recognition.

#### 5.3.3 WUCE with LOSO cross-validation

The outcomes of the WUCE cross-validation for the SEED dataset are outlined in Table 5, while those for the SEED-IV dataset are presented in Table 6.

**Table 5:** The mean accuracies (%) and standard deviations (%) of WUCE with LOSO cross-validation on the SEED database.

Methods	Pacc	Methods	Pacc
Traditional machine learning methods			
RF (Breiman, 2001)	76.42 ± 11.15*	KNN (Duda et al., 2000)	72.96 ± 12.10*
TCA (Pan et al., 2011)	77.63 ± 11.49*	CORAL (Sun et al., 2016)	82.18 ± 9.81*
SA (Fernando et al., 2013)	67.79 ± 7.43*	GFK (Gong et al., 2012)	79.28 ± 7.44*
DICE (Liang et al., 2019)	81.58 ± 7.55*	GAKT (Ding et al., 2018)	80.31 ± 6.44*
MDDD (Luo et al., 2024)	81.27 ± 5. 47*	EDPC (Dan et al., 2024)	82.31 ± 6.44*
JAGP (Peng et al., 2022)	82.13 ± 5.48*	JTSR (Peng et al., 2023)	84.53 ± 6.47*
Deep learning methods			
DAN (Long et al., 2019)	89.16 ± 7.90*	SimNet (Pinheiro, 2018)	86.88 ± 7.83*
DDC (Tzeng et al., 2014)	91.14 ± 5.61*	ADA (Philip et al., 2017)	89.13 ± 7.13*
DANN (Ganin et al., 2016)	89.45 ± 6.74*	MMD (Dino et al., 2013)	84.38 ± 12.05*
JDA-Net (Li et al., 2019b)	91.17 ± 8.11*	PARSE (Zhang et al., 2022a)	89.85 ± 5.06*
PR-PL (Zhou et al., 2024)	93.18 ± 6.55*	DADPc (Dan et al., 2025)	93.18 ± 5.40*
DLP-NNM (Wang et al., 2025)	91.52 ± 11.24*	RGNN (Zhong et al., 2020)	87.83 ± 6.21*
DAPLP (Zhong et al., 2025)	96.41 ± 2.68*	UDDA (Li et al., 2022b)	90.19 ± 10.07*
PGCN (Jin et al., 2024)	86.30 ± 5.04*	DS-AGC (Ye et al., 2025)	87.83 ± 4.21*
GCPL (Li et al., 2024)	82.51 ± 3.71*	EEGMatch (Zhou et al., 2025)	94.70 ± 4.10*
PGDLP(Ours)	**97.38** **±1.48**		

Best in bold. ^*^The performance of PGDLP is statistically significant compared with other methods at *p*-value ≤ 0.05.

**Table 6:** The mean accuracies (%) and standard deviations (%) of WUCE with LOSO cross-validation on the SEED-IV database.

Methods	Pacc	Methods	Pacc
Traditional machine learning methods			
RF (Breiman, 2001)	60.27 ± 16.36*	KNN (Duda et al., 2000)	54.18 ± 16.28*
TCA (Pan et al., 2011)	59.49 ± 12.07*	CORAL (Sun et al., 2016)	66.88 ± 14.67*
SA (Fernando et al., 2013)	56.94 ± 11.45*	GFK (Gong et al., 2012)	60.66 ± 10.00*
DICE (Liang et al., 2019)	69.68 ± 12.52*	GAKT (Ding et al., 2018)	68.77 ± 6.00*
MDDD (Luo et al., 2024)	68.81 ± 9.25*	EDPC (Dan et al., 2024)	71.39 ± 5.22*
JAGP (Peng et al., 2022)	71.72 ± 9.21*	JTSR (Peng et al., 2023)	72.43 ± 7.08*
Deep learning methods			
PARSE (Zhang et al., 2022a)	70.24 ± 8.47*	DAN (Long et al., 2019)	60.20 ± 10.20*
DDC (Tzeng et al., 2014)	68.80 ± 16.60*	MEERNet (Chen et al., 2021)	72.10 ± 14.10*
PR-PL (Zhou et al., 2024)	74.62 ± 14.15*	DADPc (Dan et al., 2025)	75.36 ± 5.13*
DLP-NNM (Wang et al., 2025)	74.27 ± 7.61*	RGNN (Zhong et al., 2020)	72.83 ± 6.25*
DAPLP (Zhong et al., 2025)	82.20 ± 9.11 -	UDDA (Li et al., 2022b)	75.96 ± 11.55*
PGCN (Jin et al., 2024)	76.10 ± 6.09*	DS-AGC (Ye et al., 2025)	**87.83** **±4.21** -
GCPL (Li et al., 2024)	82.51 ± 3.71 -	EEGMatch (Zhou et al., 2025)	72.91 ± 8.34*
PGDLP(Ours)	85.77 ± 6.82		

Best in bold. ^*^The performance of PGDLP is statistically significant compared with other methods at *p*-value ≤ 0.05.

In WUCE, individual differences among subjects are eliminated, and only environmental variations between sessions need to be addressed. As the least challenging of the four scenarios, it achieves the highest overall accuracy across contexts. As shown in Table 5, some deep learning methods have exceeded 95% accuracy, indicating that deep models can effectively capture shared features across sessions in within-subject scenarios. Algorithm 2 sets a new optimal result with an accuracy of 97.38%±7.48%, which is 2.68% higher than the best baseline. This result demonstrates that even in simple scenarios with only session variations, PGDLP's prototype-based semantic augmentation and dynamic prototype optimization can further reduce intra-class feature differences, enhance inter-class separability, and achieve near-perfect recognition accuracy. Meanwhile, it verifies the algorithm's stability in low-difficulty scenarios.

The results on the SEED-IV dataset are shown in Table 6. The DS-AGC method achieves the optimal performance with 87.83% ± 4.21%. Since the cross-session differences in the WUCE scenario primarily stem from environmental noise (e.g., device drift) rather than semantic changes, the graph contrastive learning of DS-AGC is more robust to local noise. In contrast, the prototypical semantic augmentation of PGDLP relies more heavily on global semantic consistency and exhibits weaker adaptability to local noise. The PGDLP reaches 85.77% ± 9.82% and ranks second. Despite a slight accuracy gap relative to DS-AGC in this specific test case, PGDLP achieves competitive performance and effectively alleviates the class-collision issue through adaptive prototype alignment in multi-class classification.

To verify the reliability of PGDLP's performance gains, we conducted Welch's independent samples t-tests between PGDLP and all baselines. Significance is labeled as: *(*p*-value ≤ 0.05, significant), and - (*p*≥ 0.05, no significant difference). As indicated by the asterisk notations in Tables 1–6, all traditional machine learning methods yield significantly lower classification accuracy than the proposed PGDLP method. For deep learning approaches, the vast majority of competitors achieve markedly lower accuracy than PGDLP. The PGDLP method exhibits outstanding generalization and robustness, whose performance is insensitive to variations in datasets, evaluation metrics, and experimental paradigms. Its improvement in accuracy for cross-subject EEG emotion recognition is statistically significant and not attributable to random experimental fluctuations. Although a small number of state-of-the-art (SOTA) methods achieve performance comparable to PGDLP on individual tasks, the proposed algorithm demonstrates stable, statistically verified superiority for cross-subject EEG emotion classification overall.

#### 5.3.4 CDCV results

In this section, we aim to evaluate the broad and consistent generalization capability of the proposed PGDLP method, particularly in cross-dataset emotion recognition. Essentially, achieving generalization across datasets is more formidable than cross-subject generalization, due to the significant differences between datasets.

Table 7 presents the results of cross-database cross-validation, involving a total of 6 tasks: DEAP → SEED (SI/SII/SIII) and SEED (SI/SII/SIII) → DEAP. This validation requires overcoming multiple differences across datasets, including subjects, equipment, experimental paradigms, and emotional stimuli, serving as the ultimate test of the model's ability to generalize. Consequently, the overall accuracy is significantly lower than that in intra-dataset scenarios.

**Table 7:** The mean accuracies (%) of CDCV on Seed and DEAP (SI, Session I; SII, Session II; SIII, Session III). Best in bold.

Methods	DEAP → SI	DEAP → SII	DEAP → SIII	SI → DEAP	SII → DEAP	SIII → DEAP
Traditional machine learning methods						
SA (Fernando et al., 2013)	56.69	59.33	52.28	55.61	48.90	50.02
TPT (Zheng and Lu, 2016)	58.23	60.22	55.39	60.01	51.41	52.23
GAKT (Ding et al., 2018)	60.36	61.33	59.40	59.79	52.49	54.16
MDDD (Luo et al., 2024)	61.29	62.15	61.05	61.81	56.16	56.68
DICE (Liang et al., 2019)	60.68	62.79	60.86	60.49	54.78	55.33
EDPC (Dan et al., 2024)	62.17	63.36	62.08	60.11	54.89	56.45
Deep learning methods						
DDG (Ding et al., 2018)	62.40	64.92	73.92	64.29	54.29	53.33
DDC (Tzeng et al., 2014)	60.89	62.43	69.43	62.16	52.16	50.07
DANN (Ganin et al., 2016)	61.08	62.51	72.51	63.77	53.77	52.62
DSAN (Zhu et al., 2021)	63.28	64.50	74.50	64.58	55.58	54.10
DCORAL (Sun and Saenko, 2016)	60.15	60.42	70.42	61.54	52.54	51.00
CAN (Kang et al., 2022)	64.22	65.77	75.77	**66.12**	57.12	55.39
DADPc (Dan et al., 2025)	65.83	**66.30**	75.16	65.39	57.59	58.28
PR-PL (Zhou et al., 2024)	64.71	65.22	73.34	64.60	56.06	57.38
PGDLP(Ours)	**67.59**	65.68	**75.89**	65.81	**59.27**	**59.62**

Traditional machine learning methods exhibit poor performance, with accuracy below 60% in more than half of the tasks. Although deep learning methods are generally superior to traditional methods, their effectiveness at aligning features across databases is limited. The proposed PGDLP algorithm achieves optimal performance in 4 out of the 6 tasks, namely DEAP → SI (67.59%), DEAP → SIII (75.89%), SII → DEAP (59.27%), and SIII → DEAP (59.62%), and ranks second in the remaining 2 tasks. Its overall performance is significantly superior to all baseline algorithms. Specifically, it achieves state-of-the-art accuracy of 75.89% on the DEAP → SIII cross-dataset task and outperforms all compared baseline approaches, which is 0.12% higher than the best baseline CAN; in the SIII → DEAP task, it achieves 59.62%, a 1.34% improvement.

These results demonstrate that PGDLP's prototype-based semantic augmentation and cross-domain prototype alignment mechanisms can effectively mine shared semantic information in EEG emotional features across different databases, mitigate the pronounced distributional discrepancies between databases, and thus establish it as one of the optimal solutions for cross-database EEG-based emotion recognition to date.

### 5.4 Discussion

To thoroughly examine the model's efficacy, we conduct additional evaluations to determine the influence of different configurations within the PGDLP framework.

#### 5.4.1 Ablation study

To systematically verify the contribution of each core component in the PGDLP framework to model performance, ablation experiments were conducted on the SEED dataset. Key modules, including prototypical augmentation (ProAug), intra-domain alignment (Intra-DA), and inter-domain alignment (Inter-DA), were removed, respectively. Meanwhile, control scenarios were set up, such as setting the coefficients of prototype alignment loss $\left(L_{pa}\right)$, mutual information loss $\left(L_{MI}\right)$, and consistency loss $\left(L_{\text{con}}\right)$ to 0, fixing the sample weight ${\hat{\sigma }}_{i}=1$, and fixing the clustering concentration factor ${\hat{\tau }}_{c}^{s/t}=1$. The model's recognition accuracy was tested under three typical experimental protocols: Cross-sUbject Cross-sEssion (CUCE), Cross-sUbject Single-sEssion (CUSE), and Within-sUbject Cross-sEssion (WUCE), with the results shown in Table 8.

**Table 8:** The ablation accuracy(%) of the proposed model on SEED.

Ablation Strategy	CUCE	CUSE	WUCE
PGDLP w/o ProAug	87.37 ± 5.48	92.52 ± 3.08	94.30 ± 3.84
PGDLP w/o Intra-DA	89.55 ± 3.31	93.30 ± 4.02	95.60 ± 4.49
PGDLP w/o Inter-DA	79.10 ± 5.01	84.27 ± 5.73	89.87 ± 6.17
PGDLP w/o $L_{pa}$ (λ_*A*_ = 0)	76.34 ± 5.61	81.46 ± 4.60	87.19 ± 4.73
PGDLP w/o $L_{MI}$(β = 0)	90.59 ± 4.18	94.72 ± 3.06	97.11 ± 3.51
PGDLP w/o $L_{con}$ (γ = 0)	90.34 ± 4.70	94.88 ± 3.41	96.87 ± 2.08
PGDLP w/ ${\hat{\sigma }}_{i}=1$	89.56 ± 4.33	94.85 ± 3.12	96.50 ± 2.07
PGDLP w/ ${\hat{\tau }}_{c}^{s/t}=1$	90.47 ± 3.10	96.03 ± 1.15	96.71 ± 2.27
**PGDLP(Ours)**	**91.65** **±5.16**	**96.67** **±2.25**	**97.38** **±1.48**

Best in bold.

It is found that after removing ProAug, the accuracy decreases by approximately 4% compared with the complete PGDLP model. This indicates that the semantic augmentation of target-domain features, based on the class-wise prototypes and covariance matrices of the source domain, can effectively address inter-domain semantic deficiencies, narrow the distribution gap between the source and target domains, and lay a high-quality feature foundation for subsequent label propagation and prototype alignment. Its role is particularly prominent in the CUCE scenario with significant cross-domain differences.

After removing intra-domain alignment, the model's accuracy decreases by about 2% across all three scenarios. In contrast, the model's performance drops sharply, with a maximum decrease of 12.55% after removing inter-domain alignment. This result confirms that inter-domain prototype alignment is the core to addressing the distribution shift in cross-subject/cross-session EEG signals, as it can effectively aggregate features from homogeneous prototypes in the source and target domains. In contrast, intra-domain alignment is mainly used to enhance inter-class separability within a single domain and serves as an auxiliary optimization component in cross-domain tasks.

When the prototype alignment loss coefficient λ_*A*_ = 0, the model's accuracy decreases significantly across all three scenarios, with a maximum reduction of 15.31%, the most notable performance decline among all ablation items. This demonstrates that $L_{pa}$, as the core loss that integrates intra-domain and inter-domain alignment, provides key constraints that enable the model to learn domain-invariant discriminative features by balancing intra-class compactness and inter-domain transferability. Without it, the model will lose its core ability to adapt across domains.

After removing the mutual information loss (β = 0) and the consistency loss (γ = 0), the model shows slight decreases in accuracy across the three scenarios. It can be seen that $L_{MI}$ improves the semantic information content of features by maximizing the correlation between target features and prediction results, while $L_{\text{con}}$ enhances the reliability of pseudo-labels through constraints. Both provide refined optimization for model performance but are not core modules of the framework, as their removal has a limited impact on overall performance.

When fixing the sample weight ${\hat{\sigma }}_{i}=1$ (i.e., not adjusting the sample contribution according to the uncertainty of pseudo-labels) and fixing the clustering concentration factor ${\hat{\tau }}_{c}^{s/t}=1$ (i.e., not dynamically adjusting the similarity measure according to the intra-class feature distribution), the model exhibits slight performance declines. This indicates that the adaptive sample weight based on pseudo-label entropy can reduce the interference of noisy pseudo-labels, and the dynamic clustering concentration factor can adapt to the feature distribution characteristics of different classes. Both effectively improve the model's robustness on cross-domain tasks, with a more subtle impact in scenarios such as CUSE with a single session and relatively small domain differences.

In summary, the synergistic effect of each module enables the model to achieve excellent recognition performance across diverse cross-domain scenarios.

#### 5.4.2 Effectiveness of balance factor

**Figure 2:**
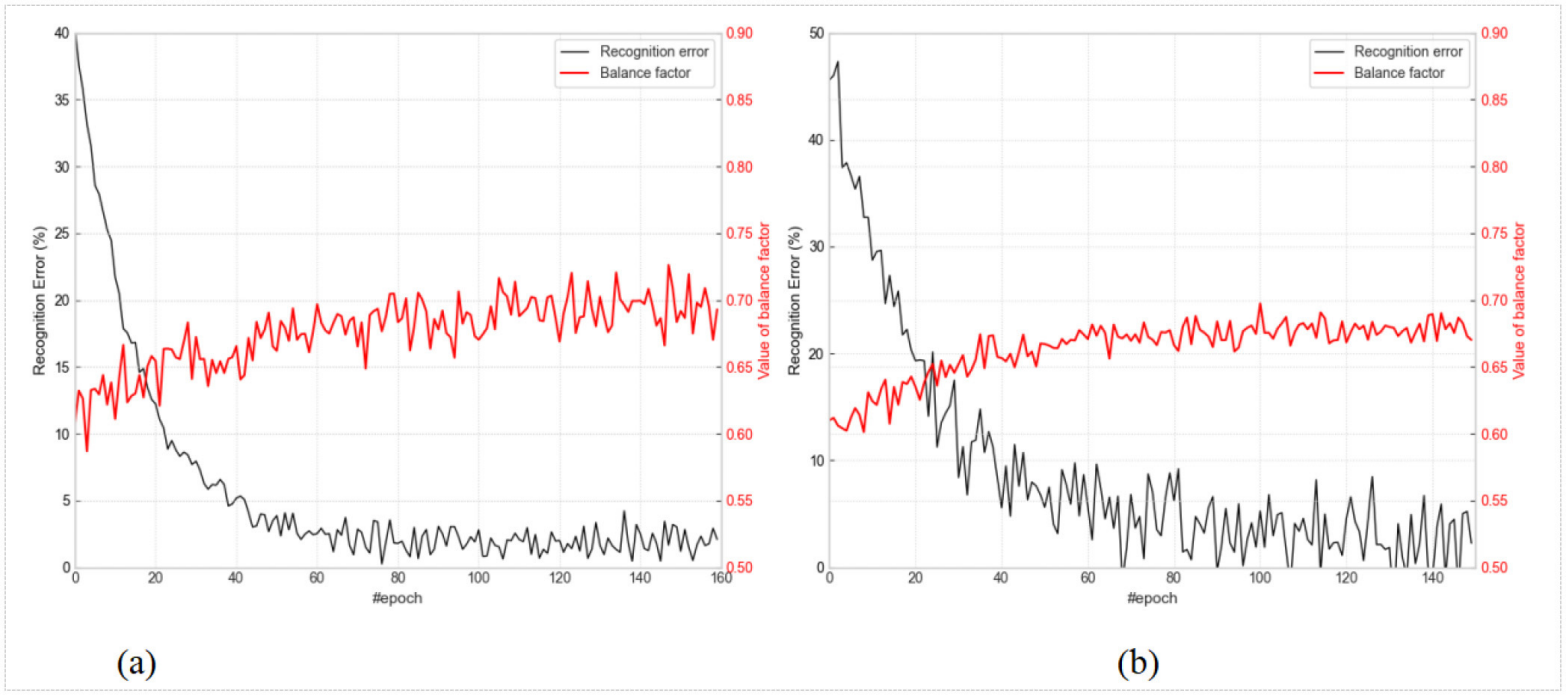
Adaptive clustering transport factor (best viewed in color). **(a)** Session 1 of WUCE. **(b)** Subject 1 of CUSE.

To verify the core role and convergence characteristics of the adaptive clustering transport factor (α) in the PGDLP framework, this study focuses on Cross-sUbject Single-sEssion (CUSE) and Within-sUbject Cross-sEssion (WUCE) tasks. By dynamically tracking the variation patterns of α values and model recognition accuracy during training, we systematically investigate its key function in balancing intra-domain discriminability and inter-domain transferability. The experimental results are illustrated in Figure 2. The left and right subfigures in Figure 2 correspond to Session 1 of the WUCE task and Subject 1 of the CUSE task, respectively. The black curve represents the model recognition error, and the red curve denotes the dynamic balance factor α.

Experimental results show that with the increase in training iterations, both the α value and model error gradually stabilize: in the WUCE scenario, α rises from the initial value of 0.6 to around 0.7 and then remains stable; in the CUSE scenario, α converges after increasing to approximately 0.67. This phenomenon indicates that PGDLP can adapt to the characteristics of different cross-domain tasks by dynamically adjusting the α value, and this adjustment process exhibits excellent convergence, providing a guarantee for the model to stably learn domain-invariant features. After convergence, the α value in the WUCE scenario (about 0.7) is higher than that in the CUSE scenario (about 0.67). This difference is highly consistent with the domain characteristics of the tasks themselves: the WUCE task only involves environmental noise differences between sessions with small inter-domain distribution shifts, thus requiring a higher α value to enhance intra-domain inter-class separability; in contrast, the CUSE task faces more significant domain shifts caused by individual differences, and thus a lower α value is needed to prioritize inter-domain transferability. This demonstrates that the adaptive αcalculation mechanism of PGDLP (based on the proportion of intra-domain and inter-domain distances) can accurately match the domain difference characteristics of different tasks, achieving dynamic balance between discriminability and transferability.

#### 5.4.3 Effect of noisy labels

**Figure 3:**
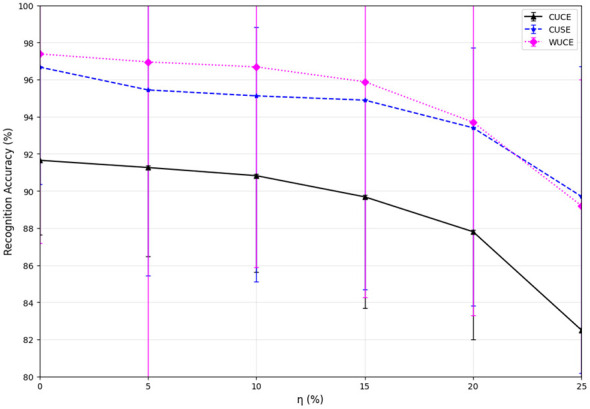
Robustness with different ratios of noise labels.

To assess the model's tolerance to label noise in practical scenarios, random noise with a proportion of η% is artificially injected into the source domain labels, and the model's generalization performance is subsequently evaluated on the unseen target domain data. Specifically, η% of the genuine labels in the source label set *Y*_*s*_ are replaced with randomly assigned labels, upon which supervised model training is implemented. Following training, the well-trained model is validated on the target domain dataset. It should be emphasized that label noise is added only to the source domain, and the target domain is used solely for performance evaluation of the trained model. In the experiments conducted, the noise ratio η% is set to five values: 5%, 10%, 15%, 20%, and 25%. The recognition accuracy of the model on the SEED dataset for each of these noise ratios is shown in Figure 3, which indicates that the model's performance degrades only slightly as the label noise ratio increases from 5% to 25%. These experimental results confirm that the proposed model PGDLP exhibits strong robustness and high insensitivity to label noise. Based on the research findings in (Zhou et al. 2024), the future research will integrate the state-of-the-art method proposed in (Jin et al. 2023), aiming to further suppress the inherent noise in EEG signals and enhance the model's stability in cross-subject emotion recognition tasks.

#### 5.4.4 Visualization and confusion matrix

**Figure 4:**
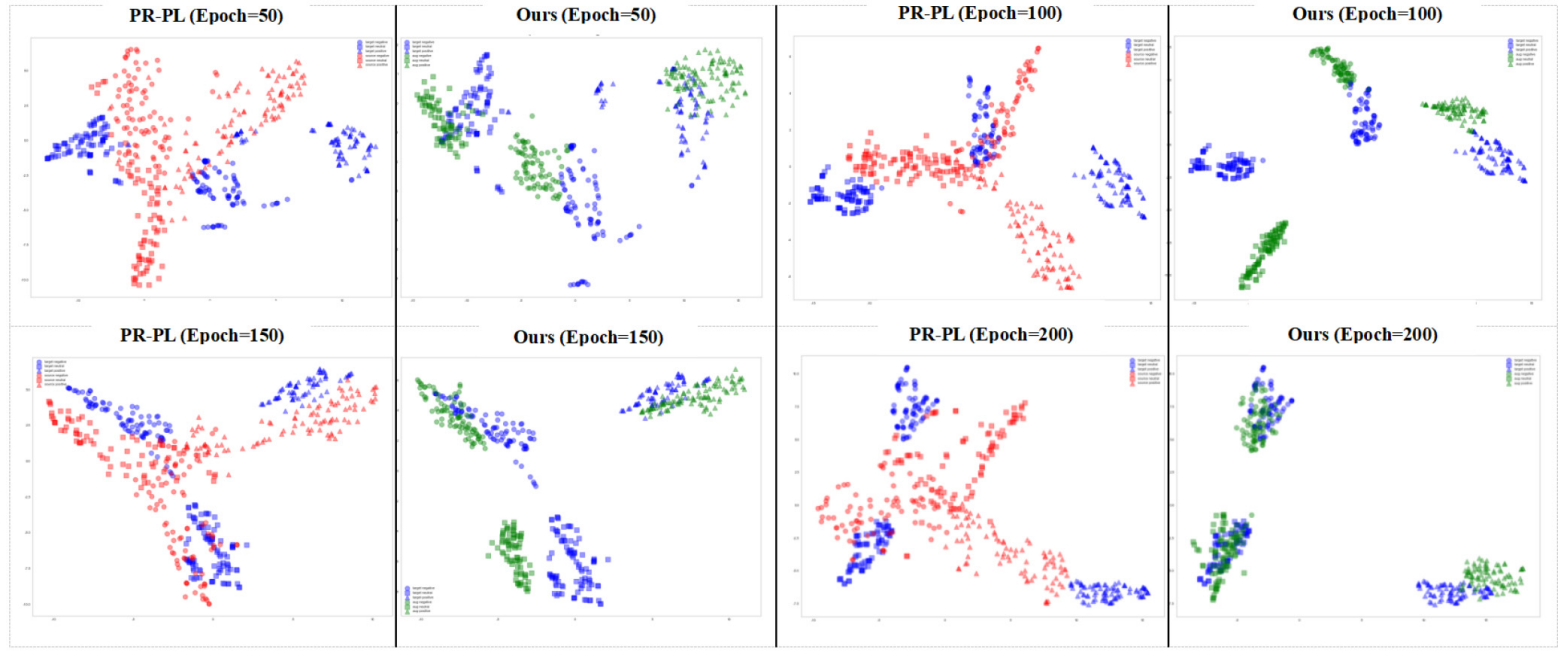
T-SNE visualization of feature alignment for Subject 1 in Session 2 on the SEED dataset with different epochs. Blue markers denote the target domain, and red and green denote the source domain in PR-PL and PGDLP, respectively. Dot, square, and triangle markers denote negative, neutral, and positive emotions, respectively.

Visualization: Figure 4 presents t-SNE (Laurens and Hinton, 2008) visualizations of feature distributions for PGDLP and PR-PL (Zhou et al., 2024) on SEED Subject 1 (Session 2) across epochs (blue markers represent the target domain, red markers denote the source domain of PR-PL, and green markers stand for the source domain of PGDLP; dots, squares, and triangles correspond to negative, neutral, and positive emotions, respectively). PGDLP shows progressive intra-class aggregation: scattered features at Epoch 50 converge into distinct, dense clusters by Epoch 200 with no cross-emotion mixing. In contrast, PR-PL retains source-target feature separation even at Epoch 200. PGDLP achieves initial inter-domain alignment at Epoch 100 and full overlap at Epoch 200, validating its efficacy in learning domain-invariant, discriminative features via prototypical augmentation and alignment.

**Figure 5:**
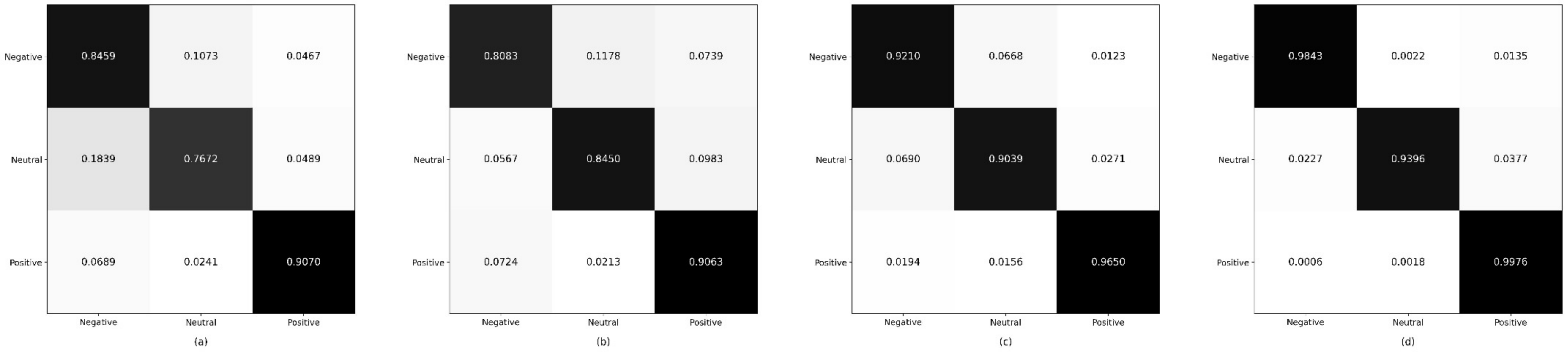
Confusion matrices of different models. **(a)** BiDANN (Li et al., 2022), **(b)** BiHDM (Li et al., 2019c), **(c)** PR-PL (Zhou et al., 2024), **(d)** PGDLP(Ours).

Confusion Matrix: To qualitatively assess the model's performance across distinct emotion categories, we visually inspected the confusion matrix of the proposed PGDLP model on the SEED dataset under the WUCE evaluation protocol, with comparisons to state-of-the-art approaches (Li et al., 2022, 2019c; Zhou et al., 2024). As illustrated in Figure 5, all compared models achieve strong recognition of positive emotions (accuracy above 90%) yet exhibit relatively inferior performance in distinguishing between negative and neutral emotions. Notably, the BiDANN model (Li et al., 2019c) achieves a neutral emotion recognition rate of 76.72%, which is below 80%. In contrast to the existing methods presented in Figures 5a–c, PGDLP demonstrates superior recognition capability, particularly for neutral and negative emotion categories. As shown in Figure 5d, the proposed model achieves recognition accuracies of 98.43%, 93.96%, and 99.76% for negative, neutral, and positive emotions, respectively. This outperforms the PR-PL model and underscores PGDLP 's robust adaptability and discriminative capacity in the target domain.

#### 5.4.5 Parameter sensitivity

**Figure 6:**
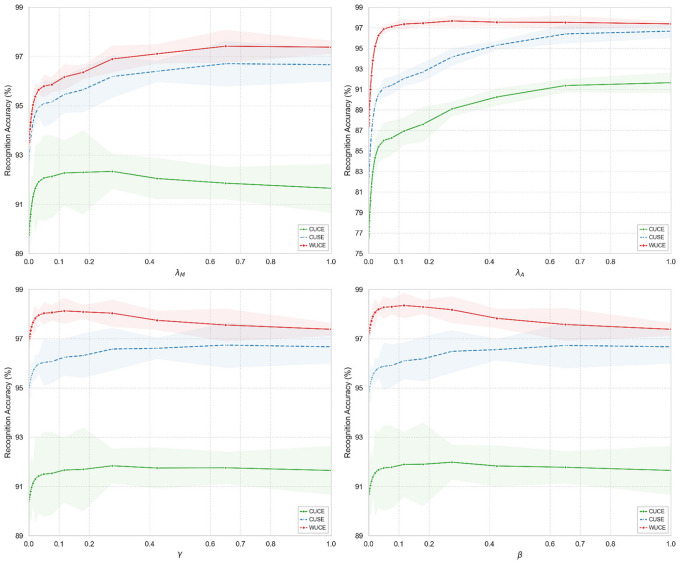
Effect of hyperparameters on SEED dataset.

To verify the stability of the PGDLP framework, we conduct experimental analysis of hyperparameter sensitivity. Exploring the impact of core hyperparameters on model performance clarifies the optimal range of parameter configurations. The experimental results are illustrated in Figure 6 (hyperparameter sensitivity).

The core hyperparameters of PGDLP include the regularization parameter λ_*M*_, prototype alignment loss coefficient λ_*A*_, consistency loss coefficient γ, and mutual information loss coefficient β. Their values directly affect the model's ability to generalize to cross-domain tasks. Experiments are conducted on the CUCE scenario of the SEED dataset to analyze the influence of each parameter on recognition accuracy:

As λ_*M*_ gradually increases from 0, the model performance shows a trend of rapid improvement first and then stabilization: when λ_*M*_ = 0, the model accuracy is at the lowest level due to the lack of smoothness constraints on label propagation; when λ_*M*_ increases to 0.65, the accuracy reaches the peak, at which point the regularization intensity precisely balances the relationship between source domain supervision information and target domain feature smoothness; after λ_*M*_ continues to increase, the accuracy does not decrease significantly, indicating that the model has strong robustness to this parameter. This result verifies the key role of λ_*M*_ in suppressing overfitting by adjusting the adjacency matrix W and the bias term b, making it particularly suitable for small-sample datasets like SEED.

The value of λ_*A*_ has a significant impact on model performance: when λ_*A*_ = 0.65, the model achieves optimal performance, and at this time, the prototype alignment loss $\left(L_{pa}\right)$ can effectively coordinate intra-domain discriminability and inter-domain transferability; when λ_*A*_ approaches 0, the model accuracy drops sharply because the absence of $L_{pa}$ leads to the loss of constraints on intra-domain compactness and inter-domain prototype alignment, resulting in an imbalance in feature learning direction; when λ_*A*_ exceeds 0.65, the performance remains stable without overfitting, indicating that this parameter has good adaptability within a wide range.

The two parameters (γ and β) have similar effects on model performance: when either parameter is 0, model accuracy decreases due to the lack of corresponding refined optimization constraints; when both parameters increase to 0.2, accuracy reaches its optimal level. At this time, $L_{\text{con}}$ enhances the reliability of pseudo-labels, and $L_{MI}$ maximizes the correlation between features and prediction results, jointly improving the quality of feature semantics; when the parameter continues to increase to 0.4 and above, the performance does not fluctuate significantly, indicating that the model is less sensitive to these two types of parameters. Stable performance can be obtained without complex parameter tuning.

#### 5.4.6 Convergence

**Figure 7:**
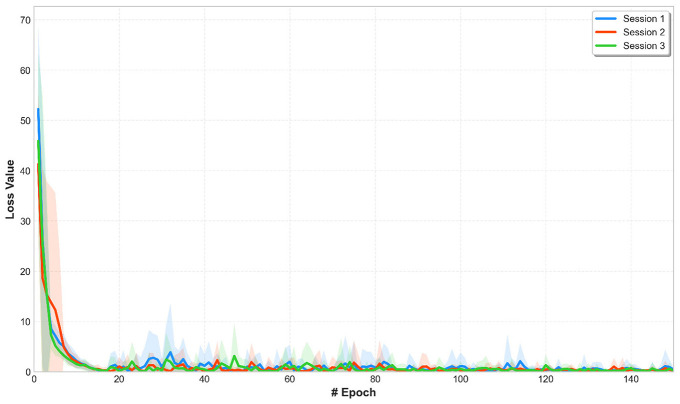
Convergence of CUSE on the target Subject 1 from the SEED dataset.

To verify the practicality of the PGDLP framework, we conduct an experimental analysis of algorithm convergence. By tracking the variation trend of model performance during training, the convergence characteristics of the algorithm are verified. The experimental results are illustrated in Figure 7 (convergence).

In all three scenarios, the objective function value of PGDLP stabilizes within 60 iterations, demonstrating the high efficiency of the framework's alternating optimization mechanism (prototype update → label propagation → network training):

- In the initial training stage (1–20 iterations), the model accuracy improves rapidly, mainly benefiting from prototypical augmentation and inter-domain alignment that quickly narrow the distribution gap between the source and target domains;
- During the 20–60 iterations, the accuracy enters a steady improvement phase, where the prototype alignment loss and refined losses jointly optimize feature discriminability;
- After 60 iterations, the model fully converges, with the fluctuation amplitude of the objective function value less than 1%, and stable performance can be obtained without additional iterations.

In summary, PGDLP exhibits the characteristics of “fast convergence speed, excellent stable performance, and strong scenario adaptability”: it can converge quickly and maintain high accuracy in low-difficulty cross-domain scenarios. However, the convergence speed slows in high-difficulty scenarios; it can ultimately achieve stable, excellent generalization performance, fully meeting the dual requirements of algorithm efficiency and reliability in practical applications.

## 6 Conclusion

To address the generalization challenges posed by the non-stationarity of EEG signals, individual differences, and semantic deficiency in cross-subject and cross-session emotion recognition, this study proposes PGDLP, a novel unsupervised domain adaptation framework that integrates prototypical semantic augmentation, prototype-graph deep label propagation, and prototypical alignment. PGDLP leverages source domain class-wise statistics for target feature semantic enhancement, constructs adaptive similarity graphs to optimize pseudo-label quality, and coordinates intra-class compactness with inter-domain transferability via dual alignment losses. Extensive evaluations on SEED, SEED-IV, and DEAP datasets across four rigorous evaluation protocols verify that PGDLP surpasses most existing state-of-the-art approaches on most test scenarios; only under the WUCE protocol of SEED-IV, our method is moderately outperformed by DS-AGC; meanwhile, PGDLP possesses strong anti-noise capability for labels and satisfactory convergence efficiency. While limitations include insufficient local noise adaptability and a lack of multimodal fusion, future work will integrate graph contrastive learning, design adaptive hyperparameter strategies, and extend to multimodal and few-shot scenarios, providing a valuable reference for unsupervised domain adaptation of physiological signals.

## Data Availability

The original contributions presented in the study are included in the article/supplementary material, further inquiries can be directed to the corresponding author.
